# Structural resolution of a small organic molecule by serial X-ray free-electron laser and electron crystallography

**DOI:** 10.1038/s41557-023-01162-9

**Published:** 2023-03-20

**Authors:** Kiyofumi Takaba, Saori Maki-Yonekura, Ichiro Inoue, Kensuke Tono, Tasuku Hamaguchi, Keisuke Kawakami, Hisashi Naitow, Tetsuya Ishikawa, Makina Yabashi, Koji Yonekura

**Affiliations:** 1 RIKEN SPring-8 Center, Sayo, Hyogo Japan; 2 Japan Synchrotron Radiation Research Institute, Sayo, Hyogo Japan; 3 Advanced Electron Microscope Development Unit, RIKEN-JEOL Collaboration Center, RIKEN Baton Zone Program, Sayo, Hyogo Japan; 4 Present Address: Institute of Multidisciplinary Research for Advanced Materials, Tohoku University, Sendai, Aoba-ku Japan

**Keywords:** Structure elucidation, Imaging techniques

## Abstract

Structure analysis of small crystals is important in areas ranging from synthetic organic chemistry to pharmaceutical and material sciences, as many compounds do not yield large crystals. Here we present the detailed characterization of the structure of an organic molecule, rhodamine-6G, determined at a resolution of 0.82 Å by an X-ray free-electron laser (XFEL). Direct comparison of this structure with that obtained by electron crystallography from the same sample batch of microcrystals shows that both methods can accurately distinguish the position of some of the hydrogen atoms, depending on the type of chemical bond in which they are involved. Variations in the distances measured by XFEL and electron diffraction reflect the expected differences in X-ray and electron scatterings. The reliability for atomic coordinates was found to be better with XFEL, but the electron beam showed a higher sensitivity to charges.

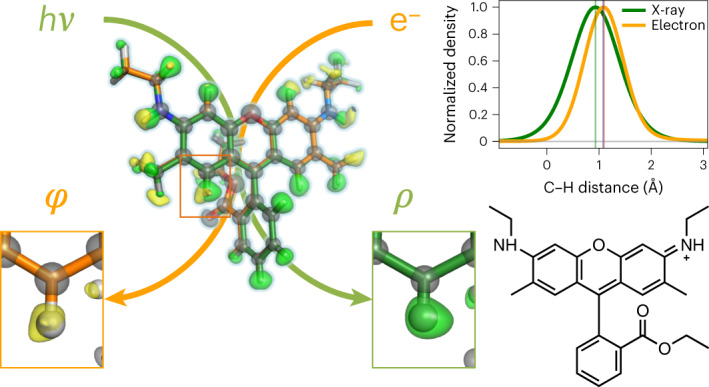

## Abstract (web summary)

The structural analysis of small crystals has remained challenging. Now, the structure of a small organic molecule, rhodamine-6G, has been resolved from microcrystals using an X-ray free-electron laser and electron diffraction. The former showed better reliability for atomic coordinates, whereas the latter was more sensitive to charges; both techniques accurately determined the position of hydrogen atoms.

## Main

Many chemical compounds do not form crystals that are large enough, or suitable enough, for conventional characterization by X-ray diffraction (XRD). However, structural analysis is important—sometimes crucial—in fields ranging from synthetic organic chemistry to pharmaceutical and material sciences, so the field of microcrystallography has been attracting increasing attention.

The atomic cross-section that defines usable sample sizes for structure determination is known to differ substantially for X-rays and electrons (X-ray, ~10^0^–10^1^ barns; electron, ~10^5^ barns)^1^. The structures of crystals that are too small for single-crystal X-ray diffraction can be resolved with atomic resolution using three-dimensional electron diffraction (3D ED)^2–4^. This technique can show the specific structure of organic molecules in small crystals^5,6^ and can also resolve the location of hydrogen atoms^7,8^. Its application, however, has remained limited by the sample thickness. X-ray free electron lasers (XFELs) generate intense and ultrafast X-ray pulses; a single pulse allows data collection before sample destruction^9,10^, and may compensate for the large differences in the atomic cross-sections of a sample. Serial crystallography with femtosecond XFEL pulses (SX/SFX) records still diffraction frames from huge numbers of small crystals^11,12^. So far, SX has mainly been applied to protein crystals, even, in some cases, resolving hydrogen densities^13,14^. However, the lattices of small molecular crystals yield fewer diffraction spots per camera frame, which leads to difficulty in the assignment of lattice indices to the diffraction spots^15,16^.

A recent study used XFEL for the determination of structures, with a resolution of 1.2–1.35 Å, from small crystals of inorganic–organic hybrid materials^17^. This approach is referred to as small-molecule (sm) SFX. The molecules in the materials characterized in that study contained several heavy atoms (Ag, Se or Te), which may have enhanced the diffraction intensity. However, the resolutions of the structures were not sufficient to obtain signals from hydrogen atoms. Through the formation of a wide range of intra- and intermolecular non-covalent bonds, hydrogen atoms contribute to a variety of local interactions, which in turn have a substantial influence on the properties of an organic molecule. Although neutron diffraction (ND) can accurately locate the positions of hydrogen nuclei^18^, ND needs much larger crystals due to there being insufficient flux from the current neutron source^19^. It is thus a challenge to use SX to investigate, from small crystals, the location of hydrogen atoms in organic compounds that do not contain heavy atoms.

X-rays are scattered by the electrons around atoms, and electrons are scattered by the Coulomb potential in their path. We believed that it would be worth comparing the structures obtained using both diffraction techniques—X-ray and electron diffraction—and evaluating their respective performances and experimental reliability. This evaluation should include hydrogen atoms; for this, the structural analysis of molecules at atomic resolution (better than 1 Å) is indispensable, but signals from hydrogen atoms are weak with both X-rays and electrons^7,8,20,21^. Only small and thin crystals are suitable for 3D ED studies, whereas XFELs need small but thicker crystals. It seemed possible that a single sample could yield a range of crystal sizes that would be useful for both techniques. In practice, small organic compounds may be the most promising for simultaneous growth of small and larger crystals.

**Fig. 1:**
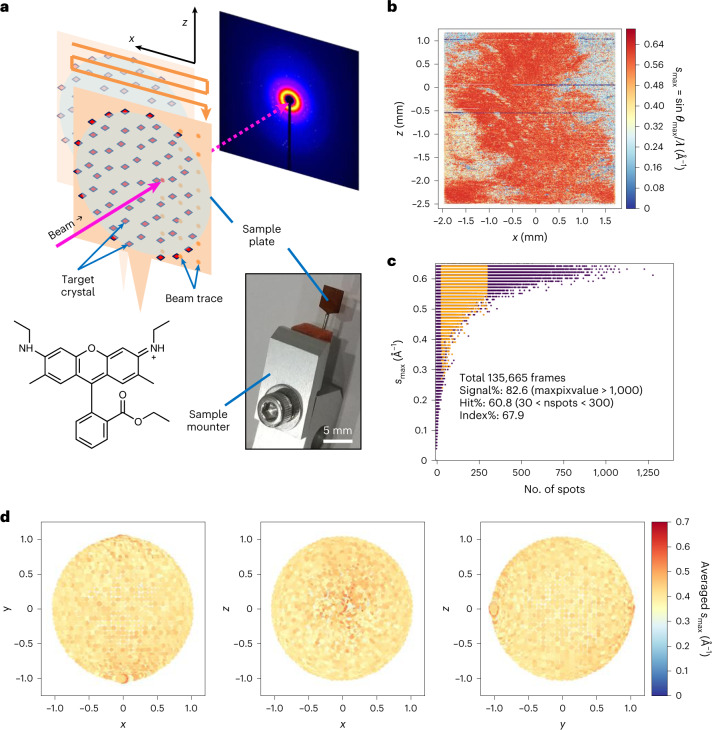
SX of rhodamine-6G crystals. **a**, Experimental set-up. Top: cartoon showing scanning of a sample plate with XFEL pluses and recording diffraction patterns on a detector. Bottom left: chemical structure of rhodamine-6G. Bottom right: sample plate fixed through a pin onto a sample–pin mounter. **b**, A typical map of the sample plate showing data-taking positions from rhodamine-6G microcrystals. The *s*_max_ of the identified diffraction spots per frame is represented by a colour dot. **c**, Plot of the spot number versus *s*_max_ = sin*θ*_max_/*λ*, where *θ*_max_ is the observed highest of half the scattering angle and *λ* is the wavelength of the X-rays, in one frame. Frames used for indexing are shown in orange. **d**, Distribution of crystal orientations over the indexed frames, as projections on the *x*–*y* (left), *x–z* (middle) and *y–z* (right) planes. The incident X-rays are along the *y* axis. The terminals of a* (a unit vector of the reciprocal lattice) are shown with coloured points. The size of each point and colour represent the number of frames contributing to the corresponding orientation and the averaged *s*_max_, respectively. Source data

In this Article we apply both electron and serial XFEL crystallography to an organic molecule, rhodamine-6G (9-[2-(ethoxycarbonyl)phenyl]-3,6-bis-(ethylamino)-2,7-dimethylxanthylium; Fig. 1a), with the samples obtained from the same sample batch containing many tiny crystals. For the use of XFEL with organic molecular crystals, we introduced a new scheme that uses a simplified sample delivery for efficient data collection as well as combining lattice parameters obtained from ED for data processing. The obtained structures show the location of hydrogen atoms.

## Results and discussion

### Structure determination at atomic resolution by SX

**Fig. 2:**
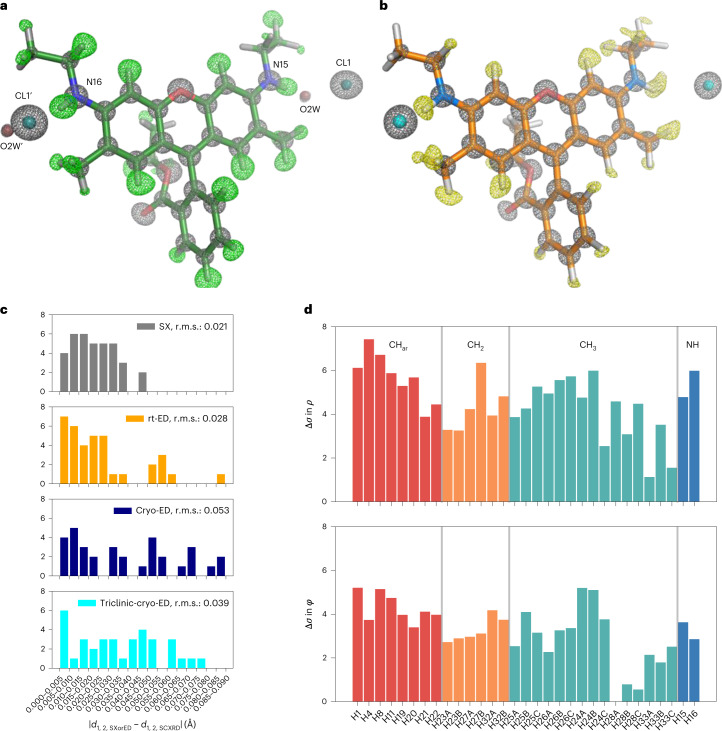
Structures of rhodamine-6G determined by SX and ED. **a**, The SX structure, containing chloride ions (CL1 and CL1′) and water molecules (O2W and O2W′). The prime symbols represent atoms related by crystallographic symmetry. **b**, The rt-ED structure. Grey nets were calculated from the observed amplitudes (*F*_o_). The hydrogen-omitting difference maps (m*F*_o_-D*F*_c_) are shown in green in **a** and yellow in **b**. Display contour levels are 3.0*σ* for all nets. The two methods reveal similar structures, including hydrogen atoms. **c**, Histograms of deviations in bond distances between the SCXRD and SX or ED structures, |*d*_SX or ED_ − *d*_SCXRD_|. Bond distances *d* are measured for 1,2-pairs. The root-mean-square (r.m.s.) values of the deviation are shown in the insets of the graph. Differences in bond lengths in comparison with the SCXRD structure are comparable for the SX and rt-ED structures. **d**, Bar plots of heights of density peaks in the hydrogen omit maps along individual hydrogen atoms. Bars are grouped by bond types, and displayed for the SX data (*ρ*) in the upper panel and for the rt-ED data (*φ*) in the lower panel. Many hydrogen densities are resolved at lower *σ* levels in the rt-ED structure than in SX. Source data

**Fig. 3:**
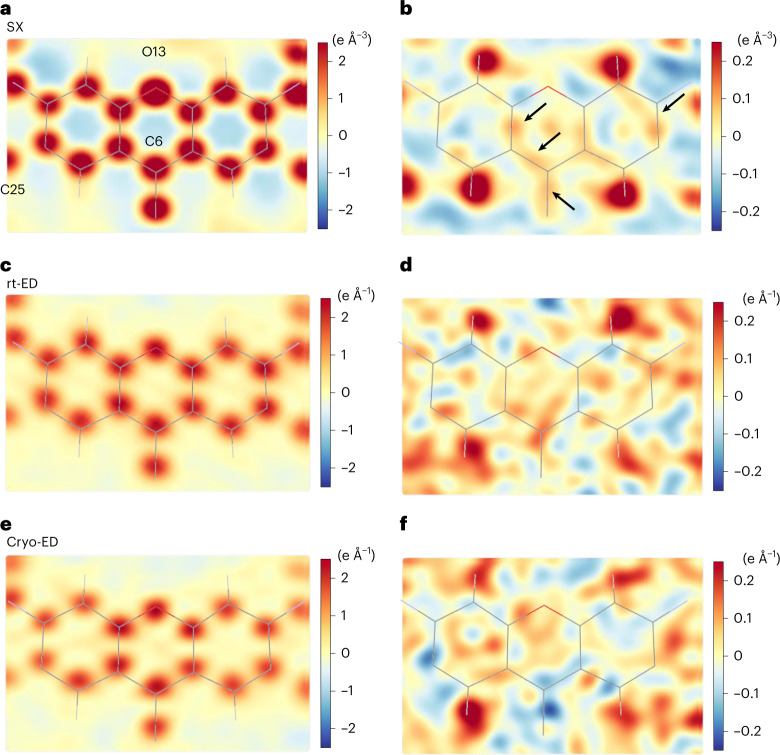
2D slices of electron density and Coulomb potential maps at the plane of the xanthene ring. **a**, A slice of the *F*_o_ map calculated from the SX data and overlaid with the model. Some atoms are labelled. **b**, A slice of the hydrogen-omitted map (m*F*_o_-D*F*_c_) from the SX. **c**,**d**, The same slices as in **a** and **b** but calculated from the rt-ED data. **e**,**f**, The same as in **c** and **d** but from the cryo-ED data. Arrows in **b** indicate residual densities that probably represent covalent electrons. The colour display is gradually changed as shown in the gradient bars to the right of each map (in corresponding units). Residual densities in the difference maps appear noisier for the ED data than for the SX data.

Microcrystals of a chloride salt of rhodamine-6G were attached on a custom-designed flat polyimide plate (4 × 4-mm^2^ square) with liquid paraffin (Supplementary Fig. 1). SX diffraction patterns were recorded at 30 Hz by 2D scanning of the sample plate (Fig. 1a), and X-ray pulses with high energy (15 keV) constantly revealed spots beyond 1 Å resolution, which were still limited by the camera size and camera distance (Fig. 1b). More than ~130,000 patterns were collected from one plate in ~1.5 h, and ‘hitting’ patterns were identified in ~65,000 frames. Compared with SX macromolecular crystallography, the number of observed diffraction spots (~20 to ~1,000) per frame appeared to be smaller (Fig. 1c), owing to the smaller lattice lengths and lower mosaicity in the crystals. Thus, assignment of lattice indices to diffraction spots in 3D was difficult without prior knowledge of the lattice parameters. We supplied the parameters obtained from an ED experiment of the same sample (see below), and this allowed for processing of the XFEL data, including partially recorded reflections, in the same way as for SX of macromolecules. The success rate of indexing from the hitting patterns was 69.2%, which is comparable to the rate for protein crystals reported previously^13,14^. The ratio of indexable patterns to the total collected ones is ~20 times better than that in a recent report on structure analysis of inorganic–organic hybrid materials at ~1.2 Å resolution by XFELs^17^. Extracted intensities were then integrated using the Monte Carlo method (Table 1). Orientations of the crystals on the sample plate were random (Fig. 1d), and all possible reflections were measured in the merged dataset (Table 1). The crystal structure was solved by the ab initio method, and a Fourier difference map obtained by subtraction of calculated structure factors, omitting hydrogen atoms, from the experimental data clearly resolved densities for hydrogen atoms (Figs. 2a and 3a,b). Residual densities between atoms in the difference map may be attributed to covalent electrons (Fig. 3b). The crystal structure confirmed that exposure to a hard XFEL pulse did not disperse away bonded hydrogen atoms in the organic molecule, even without a helium atmosphere^13^ or cryogenic cooling^14^.

**Table 1:** Summary of data taking and crystallographic data statistics

	SX	Rt-ED	Cryo-ED	Triclinic-cryo-ED
Crystal	Microcrystal	Microcrystal	Microcrystal	Re-crystallized microcrystal
Temperature	Room temp.	Room temp.	~98 K	~98 K
Wavelength (Å)	0.827	0.0197	0.0197	0.0197
No. of collected images	265,624	6,480	5,130	3,630
No. of hit images	112,871	–	–	–
No. of indexed images	78,106	5,952	3,737	2,420
No. of images used for refinement	27,908	2,852	2,727	2,057
Space group	*Pbca*	*Pbca*	*Pbca*	*P* $\bar{1}$
Unit cell (*a*/*b*/*c* in Å/*α*/*β*/*γ* in deg)	14.88/15.11/23.31	14.74/15.05/22.88	14.63/14.53/22.83	9.14/11.16/13.32/95.9/91.1/104.6
Resolution (Å)	9.65–0.82 (0.84–0.82)	11.44–0.90 (0.92–0.90)	22.83–0.90 (0.92–0.90)	13.40–0.90 (0.92–0.90)
No. of unique reflections	5,652 (556)	3,651 (243)	3,483 (217)	3,748 (240)
Completeness (%)	100.0 (100.0)	100.0 (100.0)	100.0 (100.0)	99.9 (99.2)
Multiplicity	2,688 (993.7)	81.6 (84.5)	70.8 (72.1)	15.4 (15.5)
*R*_split_ or *R*_merge_ (%)^a^	8.97 (22.77)	62.0 (372.4)	108.1 (475.3)	98.1 (293.5)
CC_1/2_ (%)^b^	98.4 (93.8)	99.6 (56.0)	99.3 (27.9)	91.7 (27.5)
*I*/σ(*I*)^c^	12.36 (3.81)	8.97 (0.83)	5.04 (0.81)	2.22 (0.55)
Resolution (Å)	9.65–0.82	11.44–0.90	11.41–0.90	13.40–0.90
*R*1 (*F* > 4σ(*F*)), *R*1 (all*F*)	0.110, 0.110	0.161, 0.200	0.206, 0.234	0.181, 0.250
*Z*^d^	8	8	8	2
*D*_calc_ (g cm^−3^)^e^	1.235	1.254	1.333	1.249
No. of atoms, C/N/O/Cl/H	28/2/3.5/1/31	28/2/3/1/31	28/2/3.5/1/31	28/2/4/1/31
No. of parameters	322	310	325	337
<*B*> for non-H atoms (Å^2^)^f^	8.36	4.25	5.82	5.12
<e.s.u.>^g^ for bond lengths (Å)	0.003	0.011	0.016	0.019
<e.s.u.> for bond angles (deg)	0.194	0.792	1.44	1.34
CCDC no.	2119567	2180418	2180417	2180416

^a^*R*_split_ is shown for SX data; *R*_merge_ is for ED data.

^b^CC_1/2_,the Pearson correlation coefficient between two half sets of intensities.^c^*I*, measured diffraction intensity.^d^*Z*, formula units in unit cell.^e^*D*_calc_, calculated density.^f^*B*, isotropic atomic displacement parameter.^g^e.s.u., estimated standard uncertainties calculated from full-matrix refinement with SHELXL^26^.

### Comparison of SX and ED structures

ED patterns were collected from the same sample batch with a parallel beam of 300-kV electrons at room (rt-ED) and cryogenic (cryo-ED) temperatures (Methods). The crystal structures were solved from merged datasets by the ab initio method, described for an organic molecule using the same electron microscope^22^. Hydrogen atoms are also resolved in the Fourier difference map (Figs. 2b and 3c–f).

**Extended Data Fig. 1:**
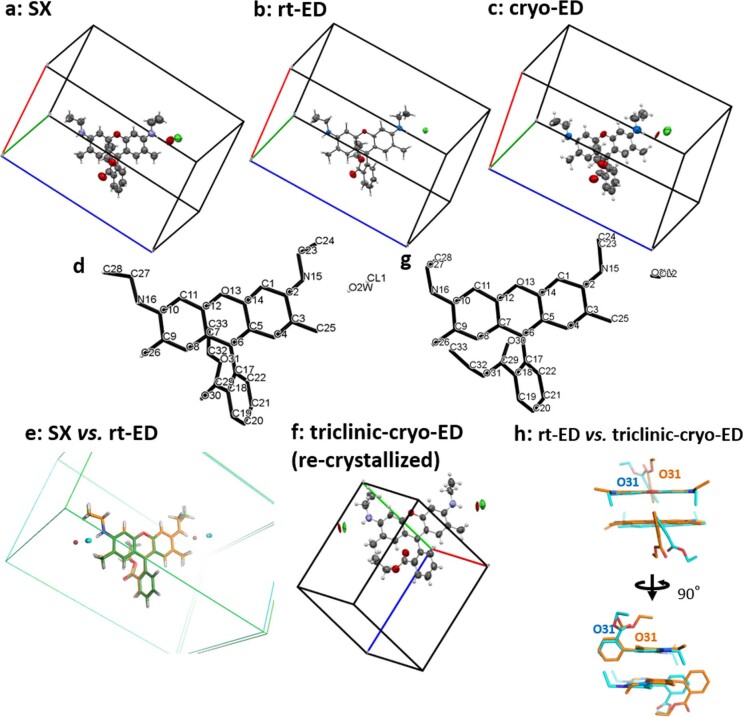
Atomic models of rhodamine-6g obtained in this study. (**a–c**) The SX model determined from the orthorhombic crystal placed in the unit cell (a), the rt-ED (b) and the cryo-ED (c) models from the orthorhombic crystal. (**d**) The atomic model in (a) with labels of non-hydrogen atoms. (**e**) Overlay of the models in (a) and (b) along with the unit cells. (**f**) The triclinic cryo-ED model from the recrystallized crystal. (**g**) The model in (f) with labels of non-hydrogen atoms. (**h**) Rhodamine-6g dimers in the orthorhombic (orange) and triclinic crystals (cyan). The rt-ED (b) and triclinic-cryo-ED (f) models are overlaid by adjusting the monomers at the top onto each other.

The structures of the rhodamine-6G monomer obtained by SX and ED are almost identical (Extended Data Fig. 1 and Supplementary Table 1), but a solvent-accessible site near the amides and the chloride is partially occupied by water in the SX and cryo-ED structures, but is empty in the rt-ED structure (Extended Data Fig. 1a–e). The rt-ED structure probably lost the water molecules in the high vacuum of the electron microscope column. Compared with the SX structure, the cell lengths along the *c* axis are smaller in both ED structures, but the cell length along the *b* axis shrinks only in the cryo-ED structure, probably upon cooling with liquid nitrogen (Table 1). Two molecules of this aromatic dye form the H-type dimer through the planes of the xanthene rings (C1 to C12 and O13; Extended Data Fig. 1d). The configuration of the dimers in this orthorhombic crystal is not identical to the triclinic crystal structure with chlorine determined previously by XRD from a single crystal (we refer to such data as SCXRD; Supplementary Discussion, Extended Data Fig. 1f–h and Supplementary Table 2). We proceeded to recrystallize the same sample following the procedure described in ref. ^23^ (Methods), obtained triclinic crystals, and determined their structure by cryo-ED. The structure (referred to as triclinic-cryo-ED) is identical to the reported triclinic crystal structure^23^ (Extended Data Fig. 1f,g, Table 1, Supplementary Table 2 and Methods). We confirm that the SX diffraction patterns could not be processed with different cell parameters, such as those in the triclinic crystal previously reported.

**Extended Data Fig. 2:**
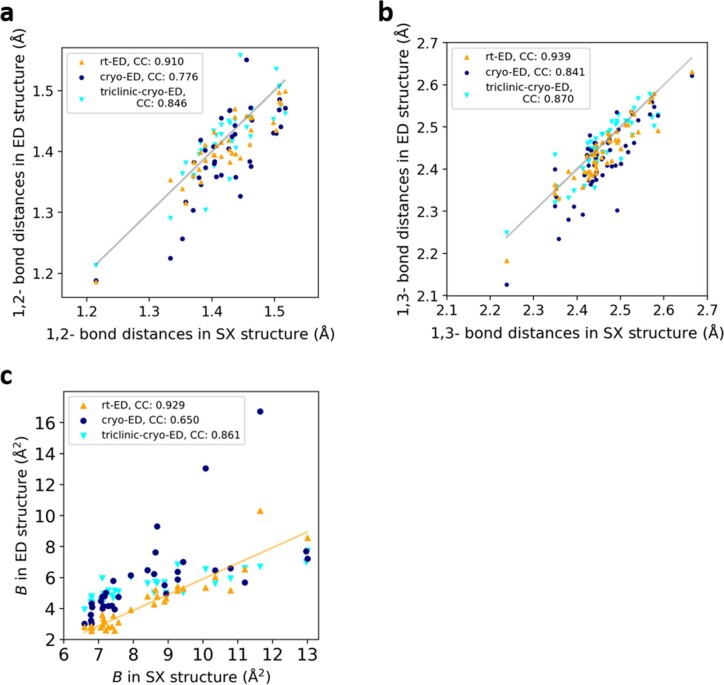
Similarities among the atomic models of the SX and ED structures. (**a–c**) Relation plots between the SX and ED structures in 1,2- (a) and 1,3- (b) bond distances for the same pair of non-hydrogen atoms and in *B*-factors (c) for the same non-hydrogen atoms. A linear regression is drawn for the rt-ED in (c). See also Supplementary Table 1 for bond lengths between non-hydrogen atoms. Correlation coefficients between the SX and ED data are also shown in the graphs. Source data

**Extended Data Fig. 3:**
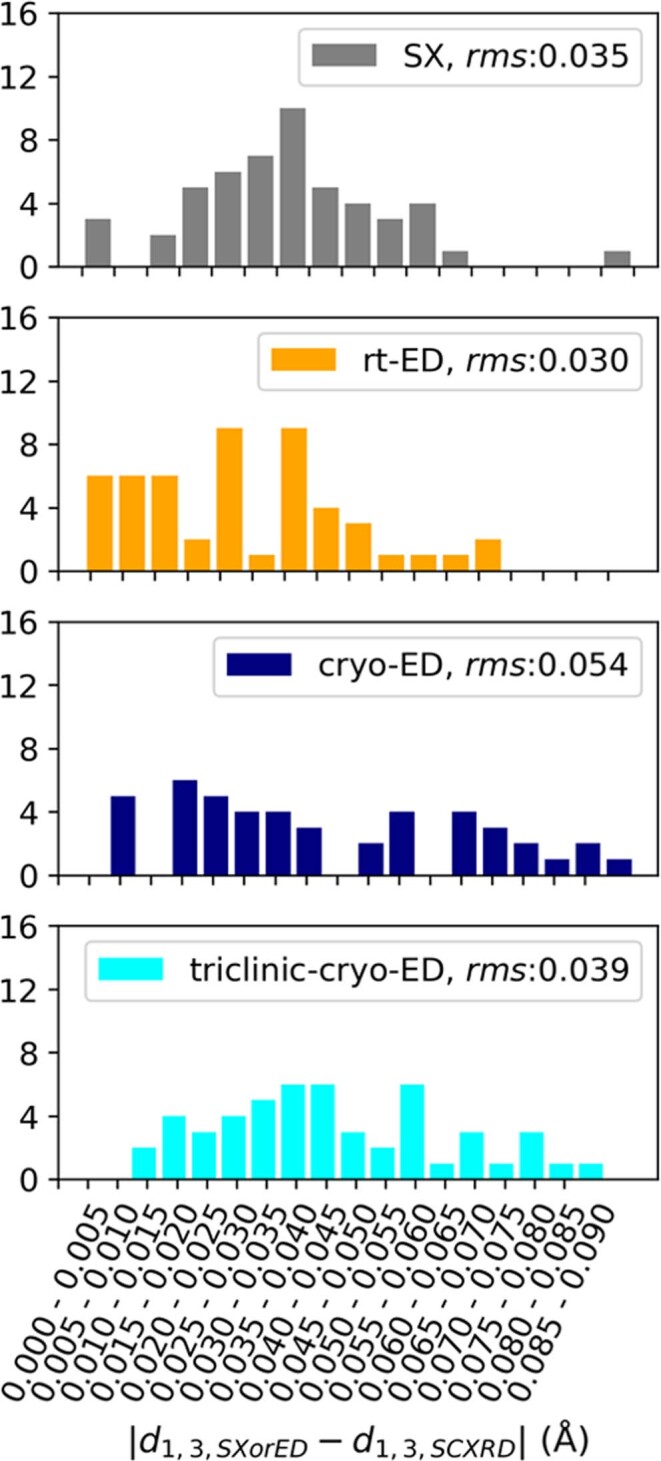
Bond deviations in the atomic models of the SX, ED structures from the reported SCXRD structure. A histogram of deviations in bond distances between the SCXRD and SX or ED structures, |*d*_SX or ED_ - *d*_SCXRD_|. Bond distances, *d*, are measured for 1,3- pairs. The root mean square (*rms*) values of the deviation are represented in the inset of the graphs. Source data

The rt-ED structure yields better data statistics and fewer deviations in bond geometry than the cryo-ED one, and the hydrogen densities were resolved at higher *σ* levels (Table 1 and Supplementary Tables 1 and 3). Radiation damage was estimated to be sufficiently small for the structural details to be maintained to a resolution of at least 0.90 Å in the rt-ED structure. The cell volume determined by ED is smaller than that determined by SX—by ~7.4% for the structure determined by cryo-ED and by ~3.2% for the rt-ED structure. The large difference in comparison with the cryo-ED structure may detract from the quality and/or homogeneity of the crystals, so, for further comparison between the ED and SX techniques, we primarily adopted the rt-ED structure. The rt-ED structure indeed shows significantly better correlations with the SX structure in terms of bond lengths and atomic displacement parameters (Extended Data Fig. 2). Additionally, when the bond lengths in the structures obtained by SX and rt-ED were compared with those in the known SCXRD structure, the differences were found to be small (Fig. 2c and Extended Data Fig. 3). We thus conclude that the positions of the non-hydrogen atoms are accurately determined by both the SX and rt-ED techniques.

Some discrepancies among the structures obtained by SX, rt-ED and SCXRD characterization may reflect different crystal packings and/or different monomer conformations. On the other hand, the estimated standard uncertainties in the bond lengths determined in the ED crystal structures are approximately two to five times larger than those in the SX structure (Supplementary Table 1). It is generally observed that the *R* factor—the discrepancy between the experimental data and the model—for an ED structure is worse than that for an XRD structure. Reflecting this, geometry errors in ED structures have also been reported to be greater^8^. Here, these tendencies in reliability for structure determination are reflected in the SX and ED structures. Hydrogen densities, particularly in alkyl groups, are also resolved at lower *σ* levels in the ED structure (Fig. 2d). The higher errors in ED structures may be attributed to factors such as a suboptimal assignment of electron scattering factors, the effects of dynamical scattering, and/or smaller numbers of units in crystals in the final datasets.

### Hydrogen densities

**Fig. 4:**
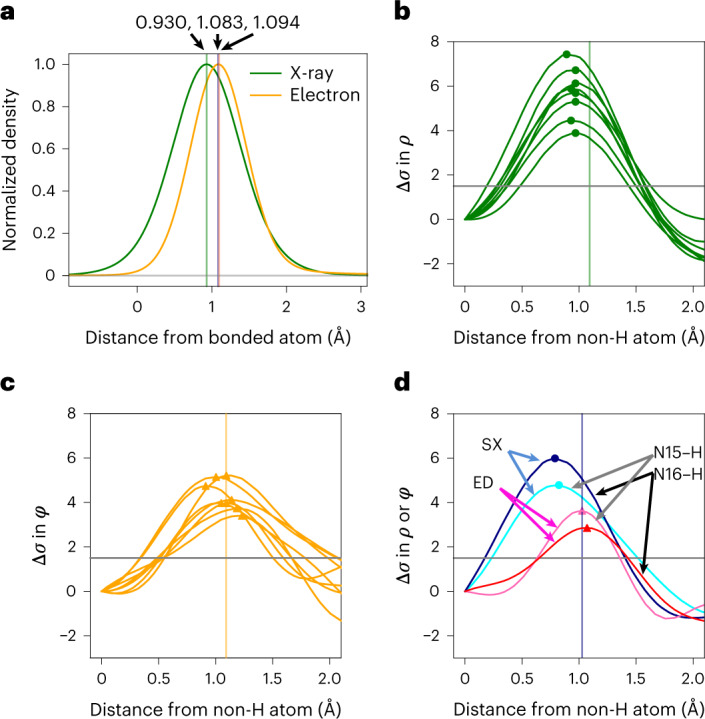
Peak positions of hydrogen densities. **a**, Theoretical curves of hydrogen density along the C–H bond. The Coulomb-potential curve in green was calculated from the electron-density curve in yellow (Methods). The peak heights are adjusted for clarity. Vertical lines represent the centre position of the curves and hydrogen nucleus (1.083 Å)^25^. **b**,**c**, Observed hydrogen density along aromatic C–H bonds in the hydrogen-omitted map in the SX (**b**) and rt-ED (**c**) structures. Small circles and triangles on the plots represent peak locations, and grey horizontal lines refer to a density threshold level of 1.5*σ*. **d**, The same plots as in **b** and **c** but for N–H bonds. The density peaks of the hydrogen atoms appear to have shorter bond lengths in the SX structure than in ED. Source data

**Extended Data Fig. 4:**
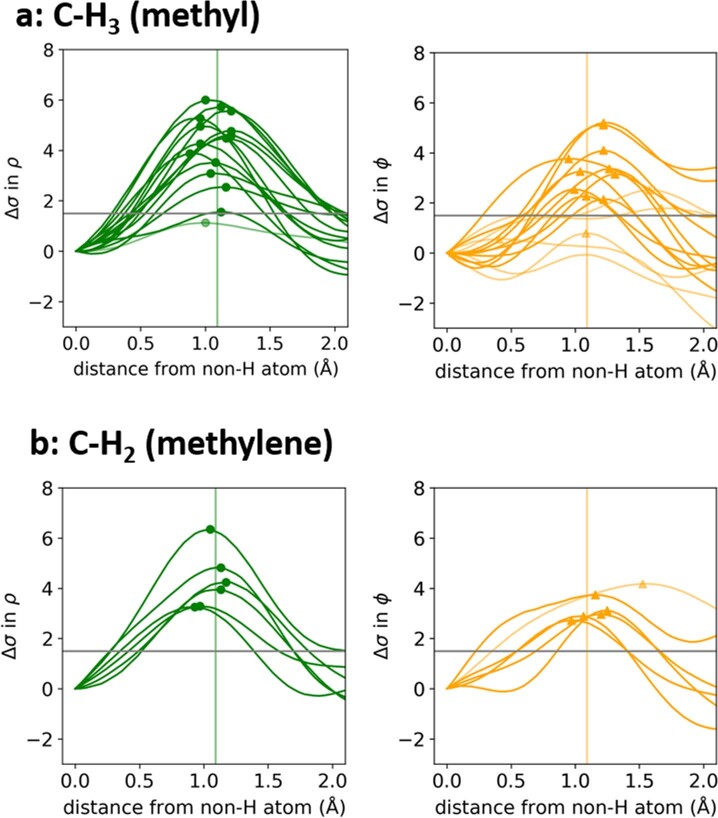
Peak positions of hydrogen densities in methyl and methylene groups. (**a**) The same density plots as in Fig. 4b, c but in C-H_3_ (methyl) bonds from the SX and rt-ED structures in green and yellow, respectively. (**b**) The same plots as in (a) but in C-H_2_ (methylene) bonds. Gray horizontal lines refer to a density level of 1.5σ, and vertical lines represent the positions of the hydrogen nuclei, obtained by ND studies^25^. Source data

Comparison of the topology of hydrogen densities in the electron-density and Coulomb-potential maps showed differences in peak positions (Fig. 2a,b). Electrons in hydrogen atoms are attracted toward bonded non-hydrogen atoms, yielding peak shifts toward the bonded atoms in the electron-density maps^18,24^. The shifts are larger in polar bonds, as the attractive force is stronger. This is in marked contrast to the nuclear density observed with ND^25^. In ED experiments, incident electrons are affected by both electrons and nuclear charges, the latter being localized in the nucleus and dominant in the Coulomb potential. Accordingly, the peak positions of hydrogen atoms are closer to the nuclei but not identical to those in the nuclear density by ND. In our theoretical calculation based on Poisson’s equation (Fig. 4a), the peak locations in the Coulomb-potential map appear slightly longer than those in ND and exhibit the opposite shift compared to that in XRD. In fact we observe that distances from the parent atom to the density peak of hydrogen appear shorter in the SX structure than in ED (cf. X-${\mathrm{H}}_{\mathrm{peak}}^{\mathrm{SX}}$ and X-${\mathrm{H}}_{\mathrm{peak}}^{\mathrm{ED}}$ in Table 2; Fig. 4b–d). Differences in these distances, ΔX–H_peak_, reveal a significant tendency to more distant positions of hydrogen density peaks in aromatic C–H bonds, whereas those in methyl and methylene C–H bonds are comparable to the standard deviation level of the peak positions (Table 2 and Extended Data Fig. 4). This observation is consistent with the hydrogen atoms of aromatic C–H bonds modelled with more of a shift toward the parent atom in XRD structures than those of methyl and methylene C–H bonds (Supplementary Table 4)^26^. These density features fundamentally arise from the polarity of C–H bonds, but also reflect the flexibility of hydrogen atoms. Motions of a hydrogen atom are more restricted along the bonding directions in the *sp*^2^-hybridized C–H bond than in the other C(*sp*^3^)–H bonds, which are revealed due to increased hydrogen visibility (Fig. 2d). Larger ΔX–H_peak_ values are measured for the amides owing to the stronger polarity in the N(*sp*^2^)–H bonds. Importantly, our SX and ED data accurately distinguish hydrogen positions in the electron density from those in Coulomb-potential maps depending on chemical bonds, as expected from the theory. This is discussed in further detail in the Supplementary Discussion.

**Table 2:** Summary of hydrogen densities

	N_all_^a^	${{{\mathrm{N}}}}_{{{{\mathrm{obs}}}}}^{{{{\mathrm{SX}}}}}$	${{{\mathrm{X}}}} - {{{\mathrm{H}}}}_{{{{\mathrm{peak}}}}}^{{{{\mathrm{SX}}}}}$ (Å)^b^	${{{\mathrm{N}}}}_{{{{\mathrm{obs}}}}}^{{{{\mathrm{ED}}}}}$	${{{\mathrm{X}}}} - {{{\mathrm{H}}}}_{{{{\mathrm{peak}}}}}^{{{{\mathrm{ED}}}}}$ (Å)	${{{\mathrm{N}}}}_{{{{\mathrm{obs}}}}}^{{{{\mathrm{SX}}\,{\mathrm{and}}\,{\mathrm{ED}}}}}$	$\Delta {{{\mathrm{X}}}} - {{{\mathrm{H}}}}_{{{{\mathrm{peak}}}}}$ (Å)
C–H_3_	15	14	1.074 (103)^c^	10	1.152 (118)	9	0.095 (175)
C–H_2_	6	6	1.064 (89)	5	1.128 (100)	5	0.077 (76)
C–H_ar_	8	8	0.949 (28)	8	1.088 (95)	8	0.139 (100)
N15–H	1	1	0.824	1	1.027	1	0.203
N16–H	1	1	0.788	1	1.070	1	0.282

^a^N_all_, ${{{\mathrm{N}}}}_{{{{\mathrm{obs}}}}}^{{{{\mathrm{SX}}}}}$, ${{{\mathrm{N}}}}_{{{{\mathrm{obs}}}}}^{{{{\mathrm{ED}}}}}$ and ${{{\mathrm{N}}}}_{{{{\mathrm{obs}}}}}^{{{{\mathrm{SX}}\,{\mathrm{and}}\,{\mathrm{ED}}}}}$ indicate the numbers of all and observed hydrogen atoms in the SX and rt-ED densities and both, respectively. Observed hydrogen densities are defined by >1.5*σ*.

^b^${{{\mathrm{X}}}} - {{{\mathrm{H}}}}_{{{{\mathrm{peak}}}}}^{{{{\mathrm{SX}}}}}$, ${{{\mathrm{X}}}} - {{{\mathrm{H}}}}_{{{{\mathrm{peak}}}}}^{{{{\mathrm{ED}}}}}$ and ΔX–H_peak_ indicate averages of observed peak positions from the parent atoms in SX and ED and differences between X-${\mathrm{H}}_{\mathrm{peak}}^{\mathrm{SX}}$ and X-${\mathrm{H}}_{\mathrm{peak}}^{\mathrm{ED}}$, respectively, respectively.

^c^Standard deviations in the same type of bond are shown in parentheses.

We then refined the rt-ED structure with the riding hydrogen model constrained with the longer bond lengths, as the resolution of the data was not sufficiently high to treat hydrogen atoms as unrestrained^8^. This yielded a drop in *R* values from 0.167 for *F*_o_ > 4*σ* and 0.206 for all *F*_o_ to 0.164 and 0.203, respectively.

### Analysis of charge

**Extended Data Fig. 5:**
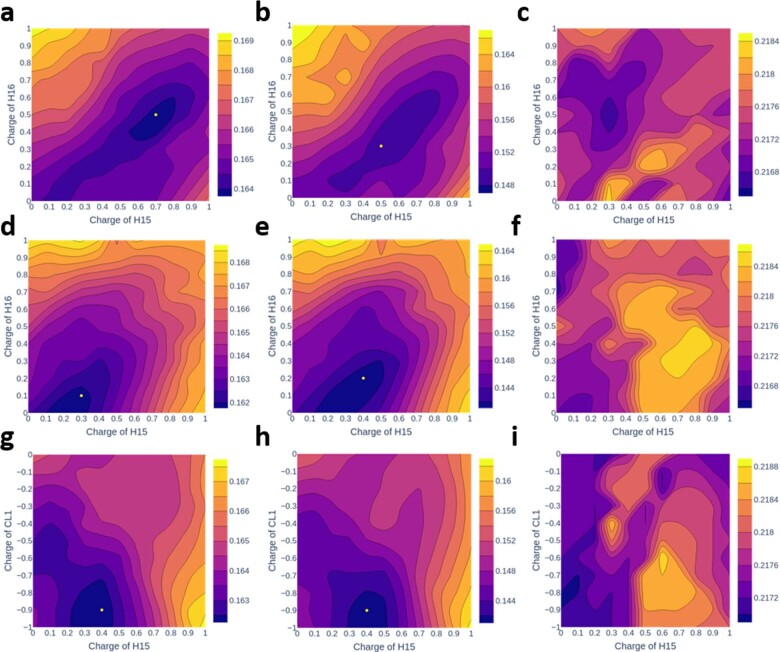
Diagrams of *R* value variations for refinement of the rt-ED structure with the charged-atom model. (**a–i**) Charge values were varied for specific atoms shown in the horizontal and vertical axes, and *R* values are represented in gradient colors according to the gradient bar on the right in each diagram. *R* values for the data with *F*_o_ > 4σ in the whole resolution shells (a, d, g), in the lowest resolution shell of *s* < 0.2 Å^−1^ (b, e, h) and in the other remaining shells (*s* ≥ 0.2 Å^−1^) (c, f, i). Amide-hydrogen atoms (H15 and H16) were exclusively charged with given values along the axes in (a – c). Given a charge of -0.9 to a chloride atom (CL1), H15 and H16 were positively charged in (d – f). Given +0.2 to H16, H15 was positively and CL1 was negatively charged in (g – i). The lowest *R* value is shown with yellow markers in a, b, d, e, g and h. All possible combinations of charges for H15, H16 and CL1 were examined as in Methods.

**Extended Data Fig. 6:**
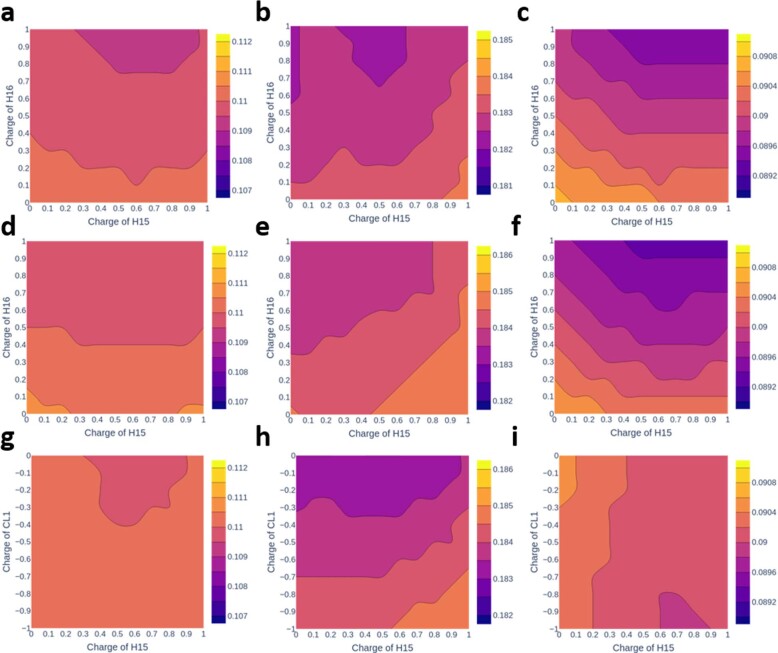
Diagrams of *R* value variations for refinement of the SX structure with the charged-atom model. (**a**–**i**) Done in the same way as in Extended Data Fig. 5, but for the SX data and model. No grid points giving *R* value minima are depicted and variations in *R* values are smaller than those for the ED data (c.f. Extended Data Fig. 5).

In theory, ED could provide charge information directly. One rhodamine-6G molecule forms a salt with a chloride ion of negative charge, and positive charge should reside around the two nitrogen atoms, N15 and N16. The ΔX–H_peak_ values are much larger in these amides than those in carbohydrates (Table 2 and Fig. 4). This indicates that both hydrogen atoms, H15 and H16, are polarized to some extent. The distances between the nitrogen atom (N15 or N16) in the amide and the chloride ion (CL1) are longer in the SX structure (3.529(3) Å between N15 and CL1; 3.433(3) Å between N16 and CL1′; Supplementary Table 5) than in the rt-ED structure (3.354(10) Å; 3.408(10) Å). These changes, and the difference in the cell length along the *c* axis, would relate to the presence of a water molecule (O2W). Thus, from the geometries around both chloride-binding sites, either of hydrogen atoms H15 and H16 would not be exclusively charged in the rt-ED structure. Indeed, *R* values are not improved when assigning a scattering factor of fully positive charge to one of the hydrogen atoms H15 or H16: 0.167 and 0.205 for *F*_o_ > 4*σ* and all *F*_o_, respectively, when H15 is given +1.0, and 0.169 and 0.207 when H16 is given +1.0 (Supplementary Table 6 and Extended Data Fig. 5a). We then changed the partial charge values in a step of 0.1 in H15, H16 and CL1 as described in ref. ^27^, and obtained a refined structure giving *R* values of 0.162 and 0.201 when assigning +0.4 to H15, +0.2 to H16 and −0.9 to CL1 (Extended Data Fig. 5d–i and Methods). The atomic coordinates were not much changed by refinement with and without assignment of charges. The same approach was tested for the SX data, but the *R* values did not improve (Extended Data Fig. 6) due to the considerably less sensitivity of X-ray scattering factors to charges^28^.

**Extended Data Fig. 7:**
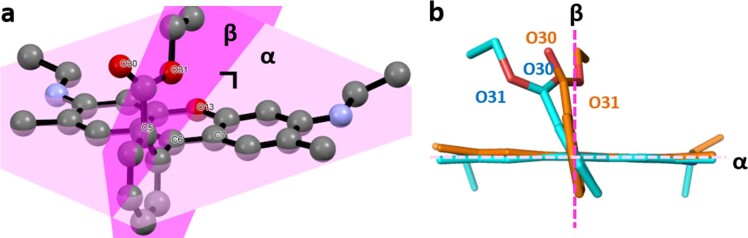
Configuration of the xanthene ring and the ethoxycabonyl tail in the SX structure of rhodamine-6g. (**a**) The plane including the xanthene ring shown in pale pink is labeled ‘α’. The plane ‘β’ in magenta is defined as a cross-section cutting through the center of the xanthene ring along O13 – C6 and perpendicular to the plane α. (**b**) A side view of (a). The ether oxygen (O31) in the ethoxycarbonyl tail faces to the xanthene-ring side and is close to the central plane β: only 0.163 Å and 0.038 Å apart from the plane in the SX- and cryo-ED structures, respectively. The distances of which are also listed in Supplementary Table 1.

Charge is evidently not localized to a particular half of the molecule, and this is reasonable for the observed configuration, where the phenolic ring at the centre of the xanthene ring is approximately perpendicular to the xanthene plane (Extended Data Fig. 1h), making both moieties nearly identical. The flipped oxycarbonyl group also appears to be consistent with this conformation, as the ether oxygen (O31) is located close to the centre of the xanthene ring (Extended Data Fig. 7 and Supplementary Table 1). This configuration contrasts to that of the triclinic-cryo-ED/SCXRD structure (Extended Data Fig. 1). Residual densities in the difference maps appear noisier for the ED data (Fig. 3d,f) than for the SX data (Fig. 3b). This theme is further discussed in the Supplementary Discussion.

## Conclusion

Intense and ultrafast XFEL pulses enabled us to record diffraction patterns from small crystals of organic molecules. We have shown the atomic-resolution structure of an organic molecule, rhodamine-6G, obtained by XFEL crystallography, and compared it with those obtained by rotational electron diffraction of the same molecule. XFEL was found to yield a structure similar to that obtained by 3D ED, but with better statistics and a more interpretable difference map, whereas 3D ED showed a higher sensitivity to charges.

Ultra-high-resolution XRD, ND and a combination of the two have been used to obtain information beyond the atomic coordinates of target molecules^29^. These studies, however, particularly those relying on ND, have been severely limited by the requirement for large crystals. The use of tiny crystals in the sample here is more practical, and we note that no special treatment was needed for either SX or ED measurements.

## Methods

### Serial X-ray crystallography

We designed a sample support composed of a polyimide flat-faced plate with an area of 4 × 4 mm^2^ and thickness of 20 μm (Protein Wave Corporation), and incorporated a metal pin attached to a tail of the plate (Fig. 1a, bottom right). Microcrystal powder of rhodamine-6G (9-[2-(ethoxycarbonyl)phenyl]-3,6-bis-(ethylamino)-2,7-dimethylxanthylium)-chloride (Tokyo Chemical Industry) was mixed with low-viscosity liquid paraffin (Nacalai Tesque), and spread over the polyimide plate. The sample was sandwiched with another plate, and held between the plates (Supplementary Fig. 1).

A prepared sample was fixed through the pin onto a specially designed sample–pin mounter and placed vertically on a sample stage of Beamline 2 at the SACLA XFEL facility^30,31^. The photon energy of the XFEL and the beam size at the sample plane were adjusted to 15.0 keV and ~1 μm, respectively. The duration and repetition rate of the pulses were 7 fs and 30 Hz, respectively. The pulse energy was ~160 μJ per pulse. The sample plate was scanned in 2D directions over the entire plate plane, the XFEL beam was exposed every 10 μm, and diffraction patterns were recorded on an MX300-HS charge-coupled device detector (Rayonix) placed 100 mm downward from the sample plane. All data collection was performed at room temperature.

CCD frames showing Bragg spots were first identified using a diffraction data-processing program, DIALS^32^ version 3.5.0, with the option *force_2d*=*True*, for *dials.find_spots*. The highest *s* = sin*θ*/*λ* (*θ* is half the scattering angle and *λ* is the wavelength of the X-rays) per frame, ${s}_{\max }$, of the identified diffraction spots was reconstructed on the sample plate with the data-taking positions (Fig. 1b). Hit frames were processed using the CrystFEL suite^33^, version 0.9.1. Frames with no pixel value >1,000 or having few (<30) or too many (>300) spots were excluded before the process, as spot indexing for these frames was less successful. Some frames taken when the beam was down (represented as blue horizontal lines in Fig. 1b) were also excluded. The selected hit frames were packed into HDF format using Python with the *h5py* package. Diffraction spots were indexed with XGANDALF^34^, Dirax^35^ and Mosflm^36^, based on lattice parameters obtained from rotational ED patterns (see below) by running *indexamajig* in the CrystFEL suite with options defined as ‘*–indexing*=*xgandalf,dirax-cell,mosflm-nolatt-nocell,mosflm-latt-nocell,mosflm-latt-cell–no-refine–int-radius*=*5,7,9–tolerance*=*5,5,5,5*’. The camera distance, beam centre position and reference lattice parameters were manually optimized by stepwise searches to improve the indexing rate. A total of 46,272 of ~136,000 frames were selected and indexed from a plate. The unit cell orientations are shown to cover all directions, without missing directions (Fig. 1d). Integrated intensities from many frames were merged by *process_hkl* in the CrystFEL suite. An initial structure was solved by ab initio phasing with SHELXT^37^. Datasets measured from two plates with success indexing rates from the hitting patterns of 67.8% and 71.4% were merged and used for structure determination. The structure was refined, and the estimated standard uncertainties of bonds and angles were calculated by SHELXL^26^. Hydrogen atoms were generated during the refinement using a riding model implemented in SHELXL, and the other restraints were removed at the end.

The distribution of crystal size was measured and estimated using an optical digital microscope (KEYENCE VHX-7000) and a function of particle analysis in OpenCV^38^ (Supplementary Fig. 1). The average diameter of crystal particles was 2.53 μm (*N* = 377), as counted from a representative image.

### Electron crystallography

Rhodamine-6G powder crystals suspended in Novec7100 (3M), an inert solvent of a hydrofluorocarbon, were spread over a 200-mesh copper grid (Maxtaform HF-34) covered with holey carbon film (Quantifoil R1.2/1.3, R1/4). This hydrofluorocarbon solvent is effective for yielding a good distribution of crystals over the grid. After drying the solution, the grid was directly cooled with liquid nitrogen, and transferred into a CRYO ARM 300 electron microscope (JEOL) operated at an accelerating voltage of 300 kV and maintained at a specimen temperature of ~300 or 98 K. A semi-automated data collection of rotational ED patterns was carried out by combined use of SerialEM^39^ and ParallEM^40,41^, using in-house scripts^22,42^. Crystals were illuminated with a parallel electron beam with a diameter of ~5.1 μm at an electron dose rate of ~0.02 e^−^ Å^−2^ s^−1^. Sequential diffraction frames per crystal were collected by continuously rotating the sample stage from −68° to 68° at a goniometer rotation speed of 1° s^−1^, and the patterns were recorded on a hardware-binned 4k × 4k-pixel array of a DE64 direct detection detector (Direct Electron). The nominal camera length was set to 800 mm and subsequently calibrated from gold sputtered on a carbon film at the end of the session.

In total, 48 and 38 rotation series for the rt and cryo datasets, respectively (numbers of datasets are written in the same manner below), were first ×2 binned, then processed with DIALS for indexing and integrating of diffraction spots. We found that the crystal form was different from that previously reported^23^. Reduced datasets were grouped and sorted by KAMO^43^, which carried out scaling and merging by using Pointless^44^, XSCALE^45^ and BLEND^46^. All merged clusters were examined for ab initio phasing by SHELXT and SHELXD^47^ and for following initial refinement by SHELXL. A merged cluster derived from 23 and 27 datasets was selected by *R*1 criteria and the number of contributed reflections. An initial structure was then refined using a model with elongated riding hydrogen distances and partially charged atoms, as described briefly in the main text and in detail below. Scaling between the experimental data and the model was adjusted using an EXTI command^48^. The geometrical restraints were finally removed, and *B* factors were kept restrained with the commands SIMU and ISOR.

The triclinic form of rhodamine-6G crystals was obtained by recrystallization as described in ref. ^23^. Briefly, the original powder of tiny crystals was dissolved in a mixed solution of methanol, ethanol and water. Crystals were obtained by drying the solution over a few days, and ED data were collected at cryogenic temperature in the manner described above. A total of 30 rotation series yielded the best cluster from 17 datasets.

### Conversion of electron density to Coulomb potential

The electron-density model of an independent hydrogen atom is expressed at a radius *r* from the centre of the electron density as

1 $$\begin{array}{*{20}{c}} {\rho \left( {{{\mathbf{r}}}} \right) = {\frac{1}{{\uppi a_0^3}}\exp \left( { - \frac{{2r}}{{a_0}}} \right)}} \end{array}$$

where *a*_0_ is the Bohr radius. The electron density is smeared by thermal motion as

2 $$\begin{array}{*{20}{c}} {\rho \left( {{{\mathbf{r}}}} \right) = {\rho \left( {{{\mathbf{r}}}} \right) \ast P\left( {{{{\mathbf{u}}}},\,{{{\mathbf{U}}}}} \right)}} \end{array}$$

where * denotes convolution, **u** defines a displacement vector from the mean position and **U** is the displacement amplitude. A probability distribution function, *P*, represents the Fourier transform of the Debye–Waller factor. The electron density can be converted to the Coulomb potential using an integrated form of Poisson’s equation:

3 $$\begin{array}{*{20}{c}} {\phi \left( {{{\mathbf{r}}}} \right) = {\frac{{Z_{{{{\mathrm{nuclear}}}}}}}{{\left| {{{{\mathbf{r}}}} - {{{\mathbf{r}}}}_{{{{\mathrm{nuclear}}}}}} \right|}} - {\int} {\frac{{\rho \left( {{{{\mathbf{r}}}}^\prime } \right)}}{{\left| {{{{\mathbf{r}}}} - {{{\mathbf{r}}}}^\prime } \right|}}} {\rm{d}}^3r^\prime }} \end{array}$$

where the nuclear charge (*Z*_nuclear_) is that for the hydrogen atom. The Coulomb potential is also converted to the smeared form, as shown in equation (2).

A 1D electron-density curve, shown in Fig. 4a, was plotted based on the model in equation (1), with a peak position of 0.930 Å, derived from SHELXL^26^. Coulomb-potential curves and the peak positions in Supplementary Table 4 were calculated using equations (1) to (3) by adopting the nuclear positions of hydrogen atoms from a ND study^25^.

### Measure of peak heights in hydrogen densities

The peak height for hydrogen atoms in the difference density maps, Δ*σ*, was measured from the estimated base density level at the corresponding bonded non-hydrogen atom in the map (Figs. 2d and 4b–d and Supplementary Table 3).

### Atomic charges around ion-binding sites

**Extended Data Fig. 8:**
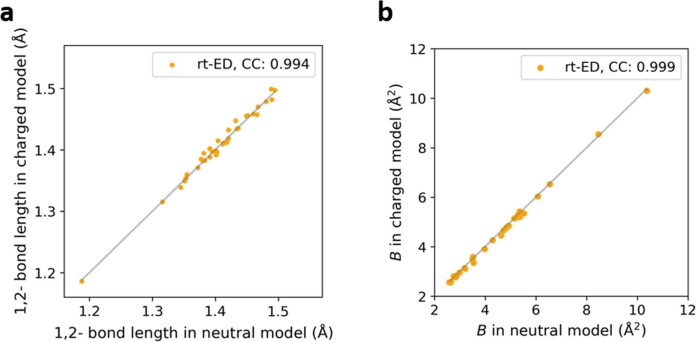
Relation plots between the neutral and charged models for the rt-ED structure. (**a**, **b**) Relation plots between the neutral and charged rt-ED structures in 1,2- bond distances (a) and in *B*-factors (b) for the same non-hydrogen atoms. Correlation coefficients are shown in the legend. Source data

Charges in pairs of amide-hydrogen and chloride atoms in rhodamine-6G were examined by a grid search over all combinations, with hydrogen positive and ≤+1.0, and chloride negative and ≥−1.0, with a step size of 0.1. Scattering factors of partially charged atoms were calculated by linear combinations of those of neutral and ionized atoms, as in a previous study^27^. All the coordinates and *B* factors were refined again for all combinations of the scattering factors. *R* values were monitored in the lowest-resolution shell (*s* < 0.2 Å^−1^), in the other remaining shells (*s* ≥ 0.2 Å^−1^) and including all the shells, as diffraction data at lower resolutions have higher sensitivity to charges. A model having charges of +0.4 for H15, +0.2 for H16 and −0.9 for CL1 provided the best *R* values for the rt-ED data in the lowest-resolution shell (Extended Data Fig. 5), but no significant improvement in *R* values was observed for the SX data. Bond lengths and *B* factors in the charged rt-ED and neutral rt-ED models were plotted for model validation (Extended Data Fig. 8), and showed no deviation between the models. Additional trials with assignment of positive or negative charge on the amide nitrogen atoms (N15 and N16) did not improve the *R* values.

### Conversion of Coulomb potential to electron density

The rt-ED experimental scattering factors were converted to X-ray structure factors by the Mott–Bethe formula^49,50^ with the obtained model structure using the GEMMI library (https://github.com/project-gemmi/gemmi). A difference Fourier map was calculated between the converted and model structure factors with neutral charges (Supplementary Fig. 2).

## Online content

Any methods, additional references, Nature Portfolio reporting summaries, source data, extended data, supplementary information, acknowledgements, peer review information; details of author contributions and competing interests; and statements of data and code availability are available at 10.1038/s41557-023-01162-9.

### Supplementary information

Supplementary Information Supplementary Discussion, Tables 1–6, Figs. 1 and 2, References and ORTEP drawing of crystal structures.

Supplementary Data 1 Crystallographic data of SX.

Supplementary Data 2 Crystallographic data of rtED.

Supplementary Data 3 Structure factors for rtED.

Supplementary Data 4 Crystallographic data of cryoED.

Supplementary Data 5 Structure factors for cryoED.

Supplementary Data 6 Crystallographic data of triclinic-cryoED.

Supplementary Data 7 Structure factors for triclinic-cryoED.

Supplementary Data 8 README document source for data processing.

Supplementary Data 9 Source Data for Supplementary Fig. 1c.

Supplementary Table 1 A workbook of Supplementary Tables 1–6.

### Source data

Source Data Fig. 1 Geometrical statistics of measured diffraction data.

Source Data Fig. 2 Geometrical statistics of molecular structure data.

Source Data Fig. 4 Geometrical features of molecular density data.

Source Data Extended Data Fig./Table 2 Geometrical statistics of molecular structure data.

Source Data Extended Data Fig./Table 3 Geometrical statistics of molecular structure data.

Source Data Extended Data Fig./Table 4 Geometrical features of molecular density data.

Source Data Extended Data Fig./Table 8 Geometrical statistics of molecular structure data.

## Data Availability

Crystallographic data have been deposited at the Cambridge Crystallographic Data Centre, under deposition nos. CCDC 2119567 (SX), 2180418 (rt-ED), 2180417 (cryo-ED) and 2180416 (triclinic-cryo-ED). Copies of the data can be obtained free of charge via https://www.ccdc.cam.ac.uk/structures/. SX image data have been deposited at the Coherent X-ray Imaging Database (CXIDB), under deposition no. 206 (https://www.cxidb.org/id-206.html). ED image data have been deposited at Zenodo (10.5281/zenodo.6684913)^51^. Please also refer to the supporting README document for using the raw image data. Source data are provided with this paper.
