# Author Correction: Structural resolution of a small organic molecule by serial X-ray free-electron laser and electron crystallography

**DOI:** 10.1038/s41557-023-01406-8

**Published:** 2023-12-13

**Authors:** Kiyofumi Takaba, Saori Maki-Yonekura, Ichiro Inoue, Kensuke Tono, Tasuku Hamaguchi, Keisuke Kawakami, Hisashi Naitow, Tetsuya Ishikawa, Makina Yabashi, Koji Yonekura

**Affiliations:** 1 RIKEN SPring-8 Center, Sayo, Hyogo Japan; 2 Japan Synchrotron Radiation Research Institute, Sayo, Hyogo Japan; 3 Advanced Electron Microscope Development Unit, RIKEN-JEOL Collaboration Center, RIKEN Baton Zone Program, Sayo, Hyogo Japan; 4 Present Address: Institute of Multidisciplinary Research for Advanced Materials, Tohoku University, Sendai, Aoba-ku Japan

**Keywords:** Structure elucidation, Imaging techniques

Correction to: *Nature Chemistry* 10.1038/s41557-023-01162-9, published online 20 March 2023.

This article was originally published under a standard Springer Nature license (© The Author(s) under exclusive license to Springer Nature Limited). It is now available as an open-access paper under a Creative Commons Attribution 4.0 International license, © The Author(s). The error has been corrected in the HTML and PDF versions of the article.

