# Supplementary material for: Structural resolution of a small organic molecule by serial X-ray free-electron laser and electron crystallography
Source: Nat Chem. 2023 Mar 20;15(4):491–7. doi: 10.1038/s41557-023-01162-9 (PMC10719108; doi:10.1038/s41557-023-01162-9)
Supplement: Supplementary file 1 — Supplementary Discussion, Tables 1–6, Figs. 1 and 2, References and ORTEP drawing of crystal structures. [file 41557_2023_1162_MOESM1_ESM.pdf]

nature chemistry

Article

https://doi.org/10.1038/s41557-023-01162-9

Structural resolution of a small organic molecule by serial X-ray free-electron laser and electron crystallography

In the format provided by the authors and unedited

Supplementary Information for

Structural resolution of a small organic molecule by serial X-ray free-electron laser and electron crystallography

Kiyofumi Takaba, Saori Maki-Yonekura, Ichiro Inoue, Kensuke Tono, Tasuku Hamaguchi, Keisuke Kawakami, Hisashi Naitow, Tetsuya Ishikawa, Makina Yabashi, and Koji Yonekura

Correspondence to: yone@spring8.or.jp

This document file includes:

Supplementary Discussion
Supplementary Tables 1 to 6
Supplementary Figure 1 to 2
Crystallographic structures
References

Supplementary Discussion

Comparison of rhodamine-6g structures

In the SX, rt-ED, cryo-ED structures, the plane of the stacked rings is approximately perpendicular to the plane of the phenolic ring (C17 to C22), which would impede resonance between rings and hinder electron transfer[52,53]. The configuration of the dimers in this orthorhombic crystal is nearly identical to that in an iodide salt crystal (CCDC#129329; Supplementary Table 2), but different from the triclinic crystal structure with chlorine determined previously by XRD from a single crystal, in which the planes of the two rings are oblique to each other rather than normal[23] (Extended Data Fig. 1, f-h). This particular dye exhibits a tendency to aggregate at higher concentrations, and hence, the effects of dimerization have been studied with respect to its photochemical properties[54,55]. A similar orientation of the H-type dimer to the SX and ED orthorhombic crystal structures was also proposed in a recent theoretical calculation[55]. However, the oxycarboxyl plane is flipped, and the configuration is different from that in the experimentally obtained structures here. The monomer conformation of the SX, rt-ED and cryo-ED structures likewise differs from the organic-solvent and metal complex structures of rhodamine-6g or its derivative (CCDC#1298021; CCDC#674356; Supplementary Table 2).

Further consideration on variations in hydrogen densities

In addition to the polarity of bonding and the motion of hydrogen atoms indicated in the main text, other factors such as smearing by truncation of information in reciprocal space, isotropic and anisotropic atomic displacement parameters, and anharmonicity along the bonding direction[18] should also be considered for quantification of the chemical characteristics at each site through electron-density and Coulomb-potential maps. The requirement for deconvolution

of these factors, however, is beyond the precision limits of the current SX and ED experiments. Practically, observations like those in this study could follow hybrid-analyses of XRD, ND and/or theoretical calculations[18,24,56]. Well-resolved hydrogen atoms, as seen in aromatic and amide groups, would be suitable candidates for further analyses.

Comparison in the electron density dimension

Residual densities in the difference maps appear noisier for the ED data (Fig. 3d, f) than for the SX data (Fig. 3b), which may represent that assignment of electron scattering factors are suboptimal as mentioned. We then converted the rt-ED experimental structure factors to X-ray structure factors by the Mott–Bethe formula[49,50] instead of view in the Coulomb-potential dimension. A difference Fourier electron density map between the converted data and calculation with neutral charges now reveals interpretable features, excess of electron on an oxygen atom but deficiency on carbon and some hydrogen atoms (Supplementary Fig. 2). This must reflect electronegativity of each atom, indicating that ED data contain information of charges. In contrast, it is known to be difficult to express electron scattering by target molecules including charges with simple theoretical scattering curves in CBED, and so structure factors are optimized against experimental CBED patterns independently of the theoretical curves[57,58,59]. This kind of treatment will need further investigation for application to 3D ED.

Supplementary Table 1 | Geometries of the atomic models of rhodamine-6g

Values in parentheses are estimated standard uncertainties derived from the full-matrix least squares refinement.

[a] Defined as a cross-section cutting through the center of the xanthene ring along O13 – C6 and perpendicular to the plane of the xanthene ring (see main text and Extended Data Fig. 7).

		d_{SX} (Å)	$d_{rt\text{-}ED}$ (Å)	$d_{cryo\text{-}ED}$ (Å)	$d_{triclinic\text{-}cryo\text{-}ED}$ (Å)
C1	C2	1.427 (3)	1.419 (11)	1.405 (16)	1.450 (18)
C1	C14	1.382 (3)	1.411 (10)	1.402 (14)	1.404 (18)
C2	C3	1.464 (3)	1.458 (10)	1.551 (14)	1.440 (19)
C2	N15	1.372 (3)	1.349 (10)	1.471 (17)	1.462 (20)
C3	C4	1.507 (3)	1.500 (12)	1.345 (14)	1.403 (19)
C3	C25	1.436 (3)	1.457 (11)	1.429 (16)	1.453 (18)
C4	C5	1.415 (2)	1.391 (10)	1.424 (14)	1.417 (18)
C5	C6	1.438 (3)	1.397 (10)	1.361 (14)	1.444 (17)
C5	C14	1.414 (3)	1.416 (11)	1.455 (15)	1.428 (17)
C6	C7	1.510 (2)	1.478 (10)	1.361 (14)	1.444 (17)
C6	C17	1.437 (3)	1.469 (10)	1.358 (13)	1.406 (18)
C7	C8	1.433 (3)	1.396 (10)	1.443 (15)	1.442 (18)
C7	C12	1.382 (3)	1.382 (11)	1.443 (15)	1.442 (18)
C8	C9	1.517 (3)	1.498 (11)	1.468 (18)	1.506 (20)
C9	C26	1.456 (3)	1.456 (10)	1.451 (15)	1.470 (19)
C9	C10	1.358 (3)	1.338 (11)	1.468 (15)	1.447 (19)
C10	N16	1.426 (3)	1.383 (10)	1.256 (14)	1.363 (18)
C10	C11	1.389 (3)	1.397 (10)	1.468 (15)	1.447 (19)
C11	C12	1.370 (2)	1.371 (9)	1.357 (13)	1.396 (17)
C12	O13	1.379 (2)	1.361 (10)	1.304 (13)	1.357 (16)
O13	C14	1.463 (4)	1.458 (12)	1.317 (14)	1.381 (18)
N15	C23	1.462 (4)	1.435 (12)	1.376 (18)	1.457 (21)
N16	C27	1.427 (3)	1.419 (11)	1.405 (16)	1.450 (18)
C17	C18	1.428 (3)	1.404 (10)	1.440 (14)	1.477 (18)
C17	C22	1.403 (3)	1.434 (10)	1.467 (15)	1.354 (21)

C18	C19	1.407 (3)	1.407 (11)	1.358 (16)	1.428 (21)
C18	C29	1.503 (3)	1.433 (10)	1.430 (15)	1.535 (20)
C19	C20	1.402 (4)	1.397 (12)	1.409 (17)	1.434 (22)
C20	C21	1.389 (5)	1.385 (12)	1.374 (19)	1.304 (23)
C21	C22	1.405 (4)	1.395 (12)	1.383 (17)	1.411 (21)
C23	C24	1.499 (5)	1.480 (14)	1.486 (19)	1.507 (24)
C27	C28	1.508 (5)	1.452 (16)	1.376 (18)	1.457 (21)
C29	O30	1.214 (3)	1.185 (9)	1.225 (13)	1.290 (18)
C29	O31	1.334 (3)	1.354 (10)	1.188 (14)	1.214 (17)
O31	C32	1.446 (6)	1.387 (15)	1.327 (24)	1.558 (27)
C32	C33	1.460 (3)	1.410 (11)	1.225 (13)	1.290 (18)

		d_{SX} (Å)	$d_{rt\text{-}ED}$ (Å)	$d_{cryo\text{-}ED}$ (Å)	$d_{triclinic\text{-}cryo\text{-}ED}$ (Å)
O30	Plane β [a]	2.701	2.712	2.676	4.114
O31	Plane β	0.163	0.038	0.512	2.253

Supplementary Table 2 | Crystal systems of rhodamine-6g compounds in Cambridge Structural Database and in this work.

[a] Atomic composition is shown for one rhodamine-6g molecule.

Unit formula[a]	Water	Ions, solvents	System	Ref.
$C_{28}H_{32}ClN_2O_{3.5}$	0.5	Cl	Orthorhombic	This work ('SX', 'cryo-ED')
$C_{28}H_{31}ClN_2O_3$	0	Cl	Orthorhombic	This work ('rt-ED')
$C_{28}H_{33}ClN_2O_4$	1	Cl	Triclinic	CCDC#164693,<br>this work ('triclinic-cryo-ED')
$C_{28}H_{33}IN_2O_4$	1	I	Orthorhombic	CCDC#129329
$C_{29}H_{34}Cl_2Zn_{0.5}N_2O_{3.5}$	0	Cl, Zn, C_2H_5OH	Triclinic	CCDC#1298021
$C_{29}H_{32}Cl_3Sn_{0.5}N_3O_3$	0	Cl, Sn, CH_3CN	Triclinic	CCDC#674356

Supplementary Table 3 | A list of hydrogen peak positions from bonded atoms. See also Fig. 4.

	X-H_{peak}, SX (Å)	peak height, SX (Δσ)	X-H_{peak}, rt-ED (Å)	peak height, rt-ED (Δσ)	ΔX-H_{peak} (Å)
H1	1.047	4.997	1.219	2.983	0.172
H4	0.891	4.795	1.080	2.599	0.189
H8	0.930	4.588	0.982	4.599	0.052
H11	0.930	3.810	1.125	3.688	0.195
H19	1.007	3.968	1.041	3.279	0.033
H20	1.007	2.918	0.997	1.415	-0.010
H21	0.891	2.889	1.040	2.860	0.148
H22	0.892	3.526	0.993	2.357	0.102
H25A	0.919	1.255	2.840	1.738	1.921
H25B	1.120	1.905	1.203	3.783	0.083
H25C	0.961	3.665	1.058	4.422	0.098
H26A	1.000	3.403	0.914	2.356	-0.086
H26B	1.200	3.503	0.770	3.084	-0.430
H26C	1.199	3.944	0.962	1.936	-0.237
H24A	0.880	2.878	1.251	1.502	0.371
H24B	0.960	0.697	0.048	0.028	-0.912
H24C	0.200	0.061	1.252	1.397	1.052
H28A	1.080	3.444	1.348	0.096	0.268
H28B	1.160	1.663	1.011	1.112	-0.149
H28C	1.240	2.541	1.443	1.090	0.202
H33A	1.039	2.413	2.309	0.507	1.270
H33B	0.800	1.396	0.000	0.000	-0.800
H33C	1.239	0.761	1.396	1.453	0.157
H23A	0.848	2.564	1.076	2.209	0.228
H23B	0.970	1.865	1.271	0.299	0.301
H27A	1.212	3.910	0.440	1.001	-0.772
H27B	1.092	5.039	0.978	0.437	-0.114
H32A	0.970	2.684	0.830	1.906	-0.139
H32B	1.132	2.008	0.684	3.503	-0.448
H15	0.789	4.096	0.775	2.355	-0.014
H16	0.752	3.516	1.047	4.484	0.295

Supplementary Table 4 | Peak positions of the hydrogen atom from the bonded atom for X-ray, neutron and electron beams.

$\mathrm{X-H_{IAM}^{XRD}}$ refers to the peak position of the hydrogen atom from the bonded non-hydrogen atom for XRD under IAM, and values in the column are adopted from ref 25; and $\mathrm{X-H_{IAM}^{ND}}$ is the same as $\mathrm{X-H_{IAM}^{XRD}}$ but determined by ND[25]. $\Delta_{\text{XRD-ND}}$ refers to difference between $\mathrm{X-H_{IAM}^{XRD}}$ and $\mathrm{X-H_{IAM}^{ND}}$. Values in $\mathrm{X-H_{IAM}^{ED}}$ were converted from those in $\mathrm{X-H_{IAM}^{XRD}}$ using the Poisson's equation for ED under IAM (see the supplementary materials). $\Delta_{\text{ED-ND}}$ and $\Delta_{\text{ED-XRD}}$ are differences between peak locations in ED ($\mathrm{X-H_{IAM}^{ED}}$) and XRD ($\mathrm{X-H_{IAM}^{ND}}$) and in ED and ND ($\mathrm{X-H_{IAM}^{XRD}}$).

	$\mathrm{X-H_{IAM}^{XRD}}$(Å)	$\mathrm{X-H_{IAM}^{ND}}$(Å)	$\Delta_{\text{XRD-ND}}$ (Å)	$\mathrm{X-H_{IAM}^{ED}}$(Å)	$\Delta_{\text{ED-ND}}$ (Å)	$\Delta_{\text{ED-XRD}}$ (Å)
$C-H_3$	0.960	1.077	-0.117	1.084	0.007	0.124
$C-H_2$	0.970	1.092	-0.122	1.109	0.017	0.139
$C-H_{aromatic}$	0.930	1.083	-0.153	1.094	0.011	0.164
N-H (CCNH)	0.860	1.027	-0.167	1.049	0.022	0.189

Supplementary Table 5 | Geometry of hydrogen bonding around amides

[a] The disposition of the three atoms is not allowed for hydrogen-bond formation.

	D – H --- A	D – H (Å)	D --- A (Å)	H --- A (Å)	D – H --- A (deg.)
SX	N15 – H15 --- CL1	0.86	3.529 (3)	2.82 (5)	141 (4)
	N16 – H16 --- CL1’	0.86	3.433 (3)	2.70 (4)	144 (3)
	N15 – H15 --- O2W	0.86	3.413 (7)	2.73 (5)	138 (4)
	N16 – H16 --- O2W’[a]	0.86	[3.696 (8)]	[3.31 (4)]	[110 (3)]
rt-ED	N15 – H15 --- CL1	1.049	3.35 (1)	2.48 (4)	140 (3)
	N16 – H16 --- CL1’	1.049	3.41 (1)	2.58 (5)	136 (3)
	N15 – H15 --- O2W	1.049	-	-	-
	N16 – H16 --- O2W’	1.049	-	-	-
cryo-ED	N15 – H15 --- CL1	1.049	3.78 (2)	3.03 (7)	129 (5)
	N16 – H16 --- CL1’	1.049	3.32 (1)	2.43 (6)	142 (4)
	N15 – H15 --- O2W	1.049	3.17 (3)	2.30 (8)	139 (5)
	N16 – H16 --- O2W’ [a]	1.049	[3.78 (3)]	[3.44 (7)]	[102 (3)]
triclinic-cryo-ED	N15 – H15 --- CL2	1.049	3.22 (2)	2.41 (8)	157 (7)
	N16 – H16 --- CL1	1.049	2.92 (2)	2.13 (8)	152 (7)
	N15 – H15 --- O2W	1.049	2.82 (3)	2.02 (8)	155 (7)
	N16 – H16 --- O1W	1.049	3.44 (2)	2.66 (8)	151 (6)

Supplementary Table 6 | Parameterization of scattering factors of partially charged atoms for structure refinement with SHELXL[26]

Element	a_1	b_1	a_2	b_2	a_3	b_3	a_4	b_4	c
$Cl^{0.1-}$	1.412	2.804	-1.793	474.3	3.026	16.62	-5.803	1899	0.1936
$Cl^{0.2-}$	1.343	2.66	-2.472	348	3.039	15.85	-7.947	1321	0.1888
$Cl^{0.3-}$	1.298	2.567	-3.234	304.5	3.045	15.36	-11.34	1198	0.1856
$Cl^{0.4-}$	1.262	2.493	-4.025	282.7	3.049	14.97	-14.82	1144	0.1829
$Cl^{0.5-}$	1.231	2.428	-4.828	269.4	3.053	14.63	-18.33	1113	0.1805
$Cl^{0.6-}$	1.203	2.369	-5.635	260.5	3.057	14.33	-21.84	1093	0.1783
$Cl^{0.7-}$	1.176	2.313	-6.444	254.1	3.06	14.06	-25.36	1078	0.1761
$Cl^{0.8-}$	1.151	2.26	-7.254	249.2	3.064	13.81	-28.87	1067	0.174
$Cl^{0.9-}$	1.127	2.209	-8.063	245.3	3.068	13.57	-32.39	1059	0.1718
$Cl^{1.0-}$	1.104	2.16	-8.871	242.1	3.071	13.34	-35.9	1052	0.1697
$H^{0.1+}$	0.1983	3.845	0.8256	193.8	0.4125	24.17	3.422	958.4	0.01586
$H^{0.2+}$	0.2464	4.74	1.762	224.9	0.5602	32.87	7.059	1022	0.01798
$H^{0.3+}$	0.2903	5.538	2.775	247.7	0.7641	41.21	10.78	1068	0.0198
$H^{0.4+}$	0.329	6.195	14.54	1101	1.007	47.99	3.833	264.8	0.02127
$H^{0.5+}$	0.364	6.742	4.913	277.5	1.273	53.13	18.33	1125	0.02246
$H^{0.6+}$	0.397	7.219	6.005	287	1.55	57.07	22.13	1143	0.02349
$H^{0.7+}$	0.4294	7.651	7.104	294.4	1.835	60.18	25.94	1157	0.02442
$H^{0.8+}$	0.4618	8.053	8.209	300.4	2.126	62.71	29.75	1169	0.02527
$H^{0.9+}$	0.4945	8.431	9.319	305.5	2.422	64.82	33.57	1178	0.02607
$H^{1.0+}$	0.5277	8.792	10.43	309.7	2.721	66.63	37.4	1186	0.02683

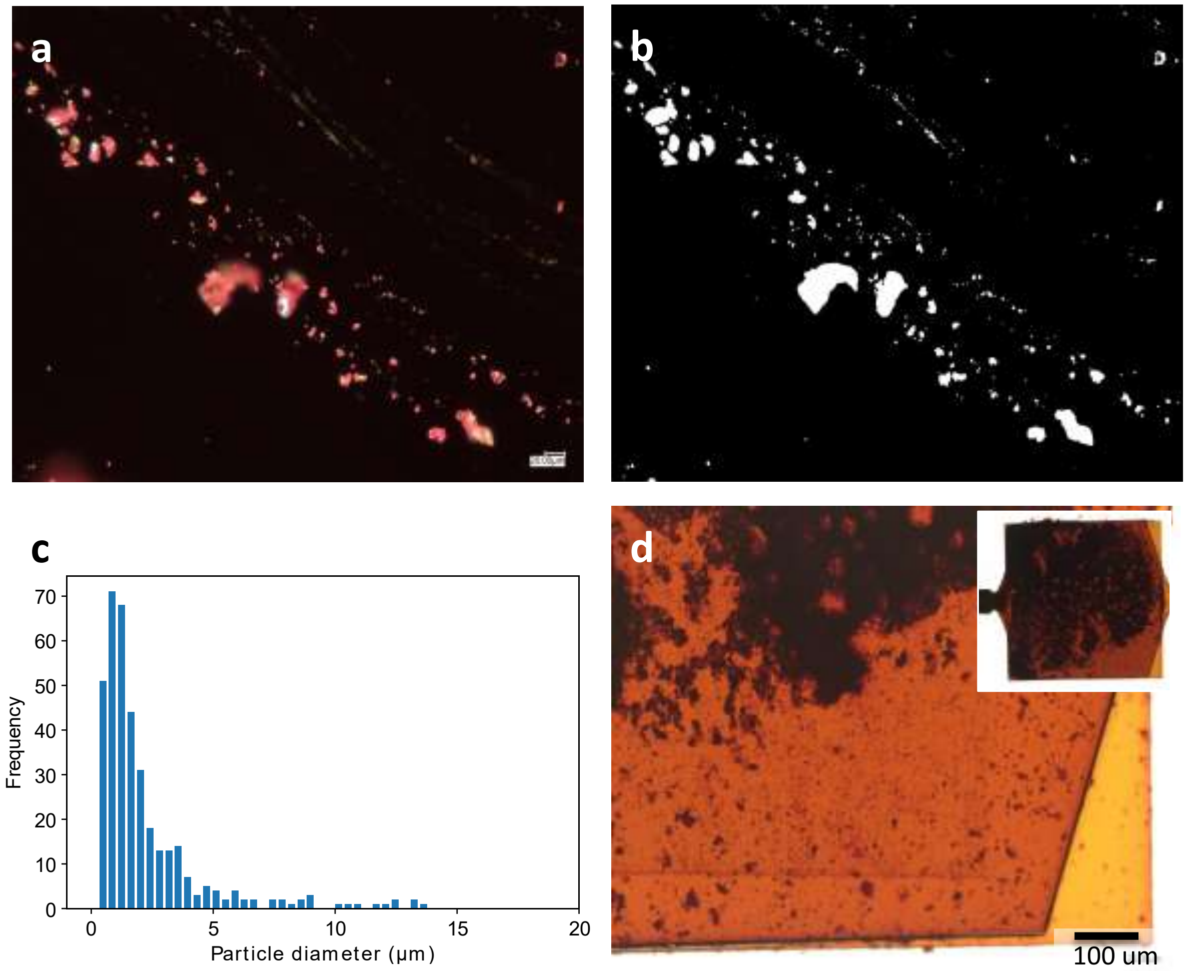

Supplementary Figure 1 | Size distribution of rhodamine-6g microcrystals. (**a**) Rhodamine-6g microcrystals spread over the slide-glass surface and observed with an optical digital microscope. (**b**) Total 377 particles, shown in white, are detected with an in-house object detection program made using OpenCV[38]. (**c**) Distribution of the detected particle diameters, which correspond to the square root of the particle area counted with (a), a representative image data. (**d**) A typical distribution of microcrystals on a polyimide plate observed after XFEL-irradiation. There are crystals appearing in dark-red in some areas and lined-up spots representing traces of beam irradiation. Location of crystals may have changed upon XFEL exposure. Inset shows the whole area of the plate (4×4 mm^2).

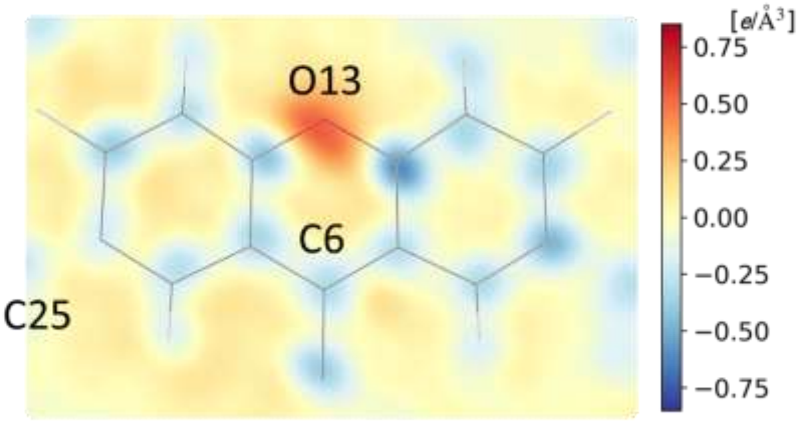

Supplementary Figure 2 | A 2D slice of a difference Fourier electron density map converted from rt-ED. Calculated between the converted F_o from the rt-ED data and F_c for X-rays with neutral charges. The color display is gradually changed as in a gradient bar at the right side.

Crystallographic structures

ORTEP drawing and our responses to the A/B-level alerts of rhodamine-6g with 'SX' data (CCDC# 2119567)

Datablock Rh6G_SX_1 - ellipsoid plot

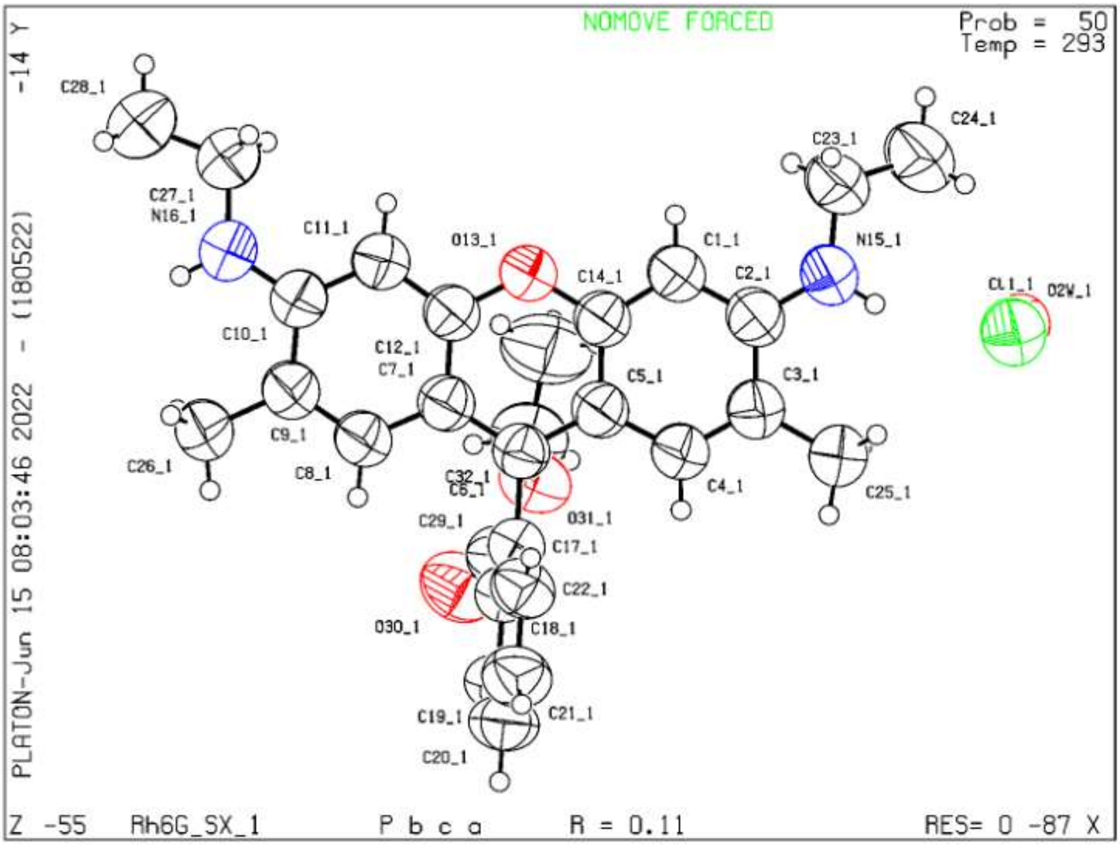

_vrf_PLAT919_Rh6G_SX_1
;
PROBLEM: Reflection # Likely Affected by the Beamstop ... 1 Check
RESPONSE: The beamstop did not affect on reflections in our experimental setups.
;
_vrf_PLAT934_Rh6G_SX_1
;
PROBLEM: Number of (Iobs-Icalc)/Sigma(W) > 10 Outliers .. 9 Check
RESPONSE: There are currently no certain reason to remove these outliers in this new method (serial data collection scheme) and we keep them.
;

ORTEP drawing and our responses to the A/B-level alerts of rhodamine-6g with ‘rt-ED’ data (CCDC# 2180418)

Datablock Rh6G_rtED_0 - ellipsoid plot

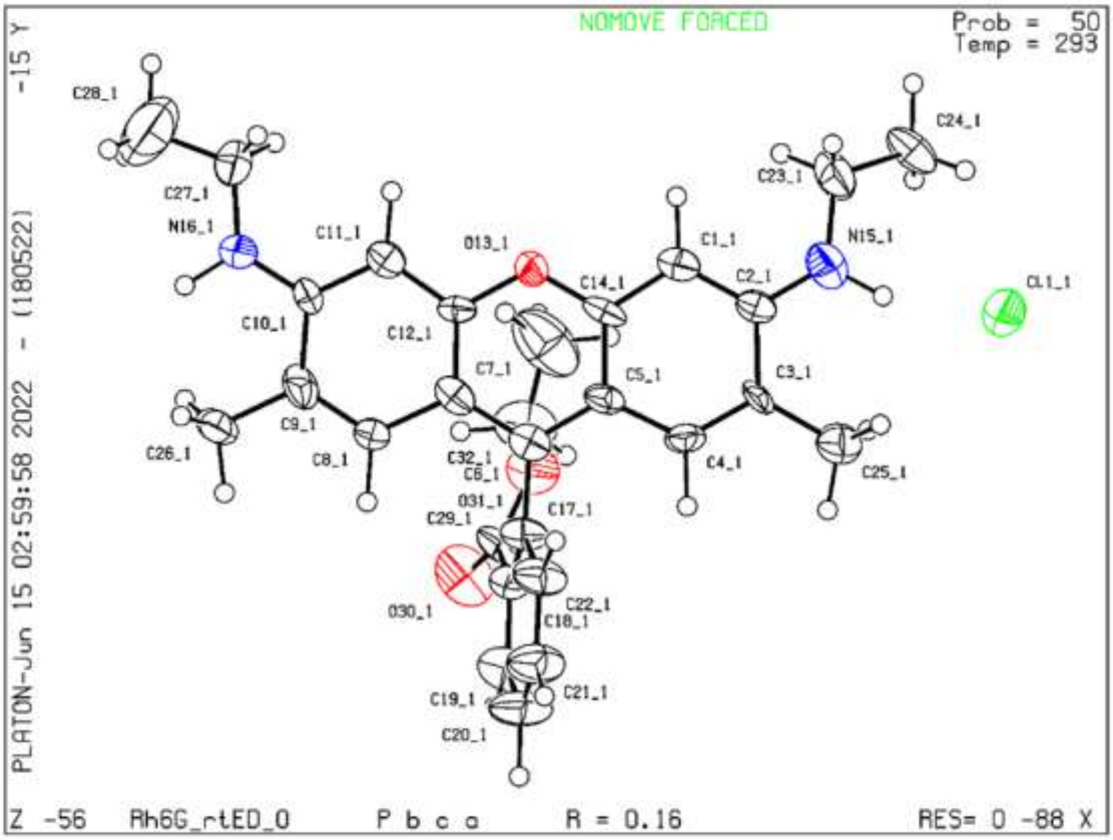

_vrf_RINTA01_Rh6G_rtED_0
;
PROBLEM: The value of Rint is greater than 0.25
RESPONSE: The value is calculated from the multiple crystals and relatively high Rint values are common for electron diffraction due to dynamical scattering.
;
_vrf_SHFSU01_Rh6G_rtED_0
;
PROBLEM: The absolute value of parameter shift to su ratio > 0.10
RESPONSE: Relatively high su values are common for electron diffraction due to dynamical scattering. Additional refinement cycles did not improve the value.
;
_vrf_PLAT020_Rh6G_rtED_0
;
PROBLEM: The Value of Rint is Greater Than 0.12 .... 0.620 Report
RESPONSE: The value is calculated from the multiple crystals and relatively high Rint values are common for electron diffraction due to dynamical scattering.
;
_vrf_PLAT770_Rh6G_rtED_0
;

PROBLEM: Suspect C-H Bond in CIF: C2_1   --H15_1   .   2.05 Ang.
RESPONSE: Not C-H Bond. This would be wrongly detected because the N-H bond is elonged to fit in Coulomb potential density.
;
_vrf_PLAT080_Rh6G_rtED_0
;
PROBLEM: Maximum Shift/Error ....   0.14 Why ?
RESPONSE: Relatively high su values are common for electron diffraction due to dynamical scattering. Additional refinement cycles did not improve the value.
;
_vrf_PLAT082_Rh6G_rtED_0
;
PROBLEM: High R1 Value ....   0.16 Report
RESPONSE: Relatively high R-values are common for electron diffraction due to dynamical scattering. Additional refinement cycles did not improve the value.
;
_vrf_PLAT084_Rh6G_rtED_0
;
PROBLEM: High wR2 Value (i.e. > 0.25) ....   0.38 Report
RESPONSE: Relatively high R-values are common for electron diffraction due to dynamical scattering. Additional refinement cycles did not improve the value.
;
_vrf_PLAT340_Rh6G_rtED_0
;
PROBLEM: Low Bond Precision on C-C Bonds ....   0.01226 Ang.
RESPONSE: Relatively low bond precision are common for electron diffraction due to dynamical scattering. Additional refinement cycles did not improve the value.
;

ORTEP drawing and our responses to the A/B-level alerts of rhodamine-6g with ‘cryo-ED’ data (CCDC# 2180417)

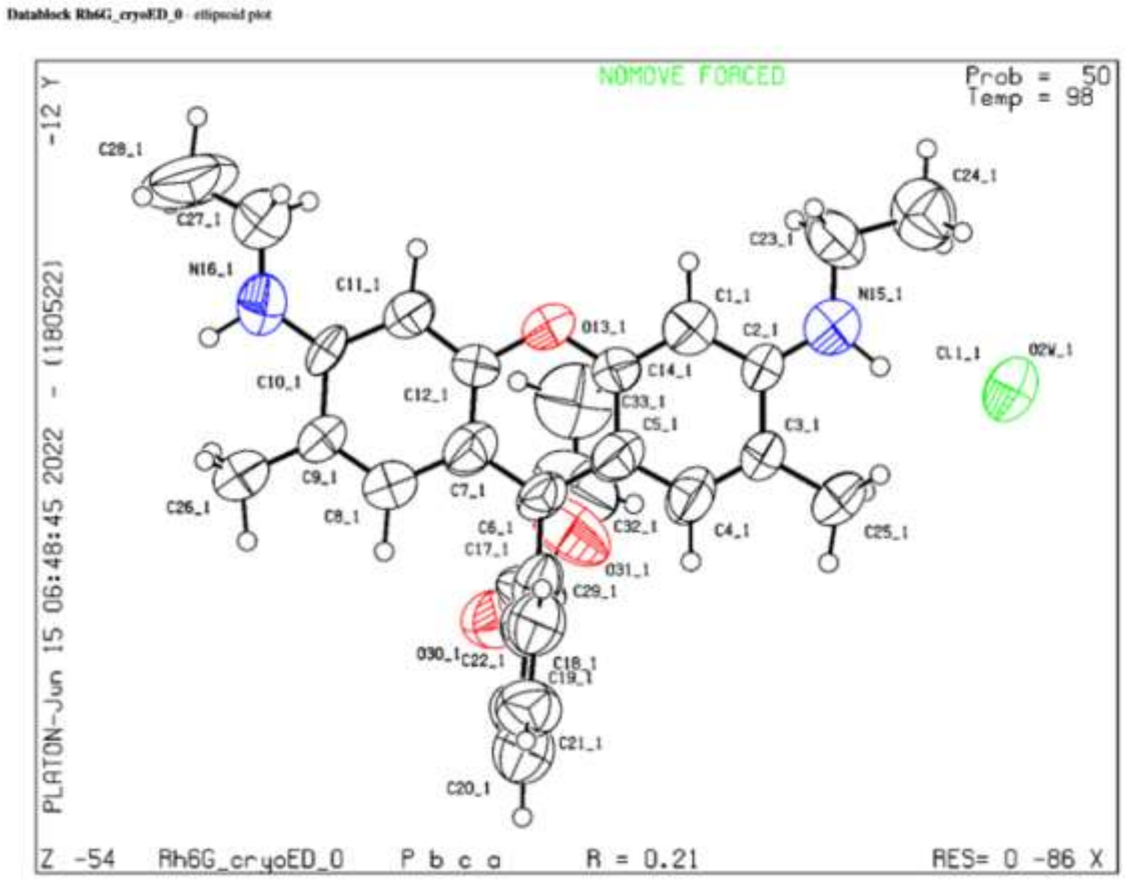

_vrf_RINTA01_Rh6G_cryoED_0
;
PROBLEM: The value of Rint is greater than 0.25
RESPONSE: The value is calculated from the multiple crystals and relatively high Rint values are common for electron diffraction due to dynamical scattering.
;
_vrf_SHFSU01_Rh6G_cryoED_0
;
PROBLEM: The absolute value of parameter shift to su ratio > 0.20
RESPONSE: Relatively high su values are common for electron diffraction due to dynamical scattering. Additional refinement cycles did not converge.
;
_vrf_PLAT020_Rh6G_cryoED_0
;
PROBLEM: The Value of Rint is Greater Than 0.12 .... 1.081 Report
RESPONSE: The value is calculated from the multiple crystals and relatively high Rint values are common for electron diffraction due to dynamical scattering.
;
_vrf_PLAT080_Rh6G_cryoED_0
;

PROBLEM: Maximum Shift/Error .... 2.38 Why ?
RESPONSE: Relatively high su values are common for electron diffraction due to dynamical scattering. Additional refinement cycles did not converge.
;
_vrf_PLAT082_Rh6G_cryoED_0
;
PROBLEM: High R1 Value .... 0.21 Report
RESPONSE: Relatively high R-values are common for electron diffraction due to dynamical scattering. Additional refinement cycles did not improve the value.
;
_vrf_PLAT770_Rh6G_cryoED_0
;
PROBLEM: Suspect C-H Bond in CIF: C10_1 --H16_1 . 1.96 Ang.
RESPONSE: Not C-H Bond. This would be wrongly detected because the N-H bond is elonged to fit in Coulomb potential density.
;
_vrf_PLAT084_Rh6G_cryoED_0
;
PROBLEM: High wR2 Value (i.e. > 0.25) .... 0.44 Report
RESPONSE: Relatively high R-values are common for electron diffraction due to dynamical scattering. Additional refinement cycles did not improve the value.
;
_vrf_PLAT234_Rh6G_cryoED_0
;
PROBLEM: Large Hirshfeld Difference O30_1 --C29_1 . 0.26 Ang.
RESPONSE: ADP values obtained from electron diffraction sometimes do not converge within the usual range for X-ray diffraction. Additional refinement cycles did not improve the value.
;
_vrf_PLAT340_Rh6G_cryoED_0
;
PROBLEM: Low Bond Precision on C-C Bonds .... 0.01922 Ang.
RESPONSE: Relatively low bond precision are common for electron diffraction due to dynamical scattering. Additional refinement cycles did not improve the value.
;

ORTEP drawing and our responses to the A/B-level alerts of rhodamine-6g with ‘triclinic-cryo-ED’ data (CCDC# 2180416)

Datablock Rh6G_tricliniccryoED_0 - ellipsoid plot

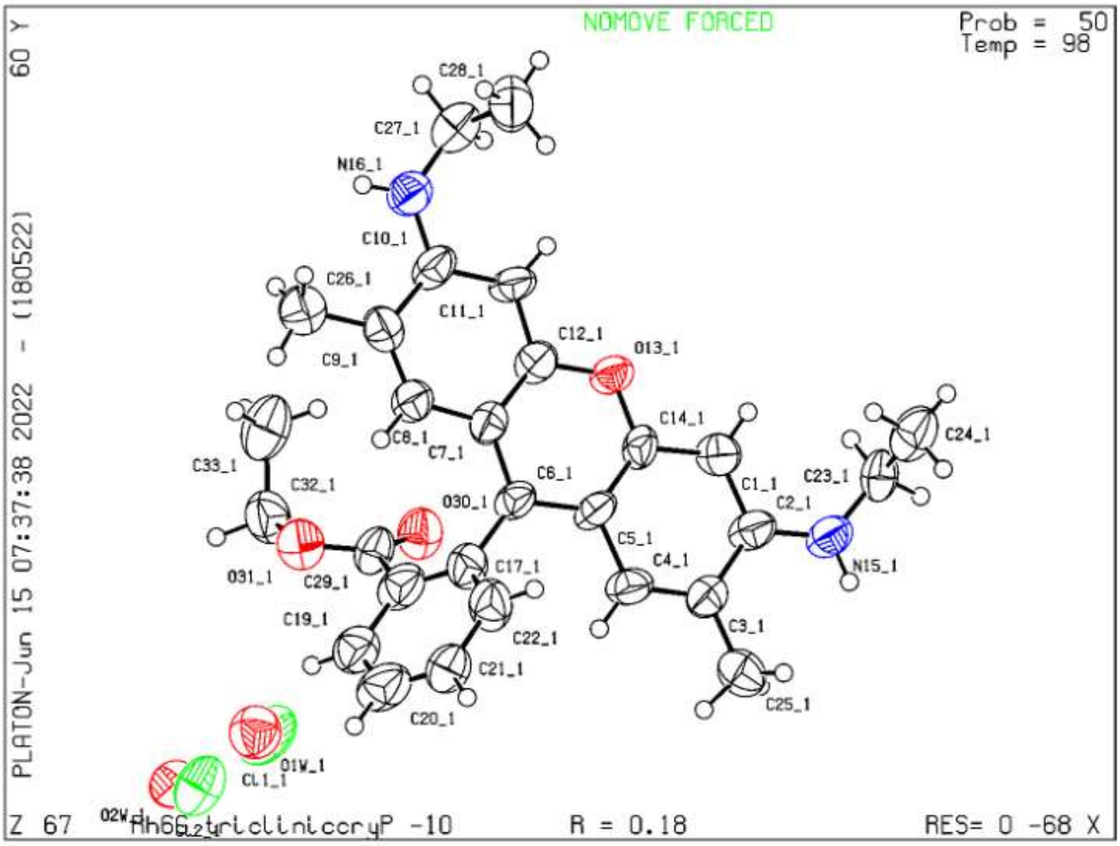

_vrf_RINTA01_Rh6G_tricliniccryoED_0
;
PROBLEM: The value of Rint is greater than 0.25
RESPONSE: The value is calculated from the multiple crystals and relatively high Rint values are common for electron diffraction due to dynamical scattering.
;
_vrf_PLAT020_Rh6G_tricliniccryoED_0
;
PROBLEM: The Value of Rint is Greater Than 0.12 .... 0.981 Report
RESPONSE: The value is calculated from the multiple crystals and relatively high Rint values are common for electron diffraction due to dynamical scattering.
;
_vrf_PLAT082_Rh6G_tricliniccryoED_0
;
PROBLEM: High R1 Value .... 0.18 Report
RESPONSE: Relatively high R-values are common for electron diffraction due to dynamical scattering. Additional refinement cycles did not improve the value.
;
_vrf_PLAT084_Rh6G_tricliniccryoED_0

;
PROBLEM: High wR2 Value (i.e. > 0.25) .... 0.44 Report
RESPONSE: Relatively high R-values are common for electron diffraction due to dynamical scattering. Additional refinement cycles did not improve the value.
;
_vrf_PLAT234_Rh6G_tricliniccryoED_0
;
PROBLEM: Large Hirshfeld Difference C20_1 --C21_1 . 0.26 Ang.
RESPONSE: ADP values obtained from electron diffraction sometimes do not converge within the usual range for X-ray diffraction. Additional refinement cycles did not improve the value.
;
_vrf_PLAT340_Rh6G_tricliniccryoED_0
;
PROBLEM: Low Bond Precision on C-C Bonds .... 0.02093 Ang.
RESPONSE: Relatively low bond precision are common for electron diffraction due to dynamical scattering. Additional refinement cycles did not improve the value.
;

References

52. Koide, Y., Urano, Y., Hanaoka, K., Terai, T. & Nagano, T. Evolution of Group 14 rhodamines as platforms for near-infrared fluorescence probes utilizing photoinduced electron transfer. *ACS Chem. Biol.* **6**, 600–608 (2011).
53. Kjær, C., et al. Gas-phase action and fluorescence spectroscopy of mass-selected fluorescein monoanions and two derivatives. *Phys. Chem. Chem. Phys.* **22**, 9210–9215 (2020).
54. Bujdák, J., Iyi, N. & Sasai, R. Spectral properties, formation of dye molecular aggregates, and reactions in rhodamine 6G/layered silicate dispersions. *J. Phys. Chem. B* **108**, 4470–4477 (2004).
55. Chapman, M. & Euler, W. B. Rhodamine 6G structural changes in water/ethanol mixed solvent. *J. Fluoresc.* **28**, 1431–1437 (2018).
56. Madsen, A. Ø., Mason, S., & Larsen, S. A neutron diffraction study of xylitol: Derivation of mean square internal vibrations for H atoms from a rigid-body description. *Acta Crystallogr. Sect. B Struct. Sci.* **59**, 653–663 (2003).
57. Zuo, J. M., Kim, M., O'Keeffe, M. & Spence, J. C. H. Direct observation of d-orbital holes and Cu-Cu bonding in Cu2O. *Nature* **401**, 49–52 (1999).
58. Ogata, Y., Tsuda, K., Tanaka, M. Determination of the electrostatic potential and electron density of silicon using convergent-beam electron diffraction. *Acta Crystallogr. Sect. A Found. Adv.* **64**, (2008).
59. Nakashima, P. N. H., Smith, A. E., Etheridge, J. & Muddle, B. C. The bonding electron density in aluminum. *Science* **331**, 1583–1586 (2011).
